# The MyoPulser field stimulator, a do it yourself programmable electronic pacemaker for contracting cells and tissues

**DOI:** 10.1038/s41598-023-29145-3

**Published:** 2023-02-11

**Authors:** Christiane Ott, Tobias Jung

**Affiliations:** 1 Department of Molecular Toxicology, German Institute of Human Nutrition Potsdam-Rehbruecke (DIfE), Arthur-Scheunert-Allee 114-116, 14558 Nuthetal, Germany; 2 German Center for Cardiovascular Research (DZHK), 10117 Berlin, Germany

**Keywords:** Biophysics, Physiology, Cardiology

## Abstract

After providing the free software MYOCYTER that analyzes a large amount of data from videos of contracting cells, tissues or organs, we now present an “*Arduino*”-based programmable, customizable and cost-effective electronic pacemaker (“MyoPulser”) that triggers contraction by electric stimulation of the sample at arbitrary frequencies. In this work, construction, functions and application of the MyoPulser are explained in detail, the electronic pacemaker is also tested on isolated cardiomyocytes and HT22-cells to quantify biological effects of pacing. The device enables the user to select between different pulse types (monophasic, alternating, bi- and polyphasic) adjust the length of an applied pulse (1–200 ms), the gap between two consecutive pulses (20–2000 ms), application of irregular pulses with random length and gaps (simulation of arrhythmia) in a user-defined range, as well as manual pulsing, while extensive data are recorded for every single pulse during the experiment. Electrostimulation of isolated B6 cardiomyocytes showed very little deviation of the observed cellular contraction from the applied pulse settings of the device, while the carbon electrodes used proved to be biologically inert in long-term experiments. Due to the open source code and the expandable setup, the MyoPulser can be easily adapted to even highly specific requirements and together with the software MYOCYTER it represents a complete cardiomyophysiological measuring station.

## Introduction

To analyze and quantify spontaneous or induced cellular contractions, we have developed the software MYOCYTER^1^ in the first part of our work. This program extracts an unprecedented number of parameters from high speed videos of contracting cells^2–5^.

In order to be able to study cell movements under electrical stimulation of different frequencies, we provide in this work the exact instructions for the corresponding hardware, a high precision but cost-effective electronic pacemaker (“MyoPulser”). Thus, this work is the practical part of our project to provide an open pacing system for (cardio-)myophysiological research.

Main aim of this work was the development of a low-cost electronic pacemaker for contracting cells and tissues that can be assembled, programmed and individually customized by anyone, providing the same or even more functions as commercial field stimulators.

With MYOCYTER and MyoPulser researchers have an open and complete soft- and hardware solution, a powerful tool for the analysis of any contracting cell type or tissue. To our knowledge, this is the first time such a device is published as do it yourself instruction. Construction and material costs are in the range of about $55–100 (depending on the microcontroller^6–8^ used). Due to the open source code and expandable, inexpensive hardware, MyoPulser can be easily adapted to virtually any needs for even highly specialized individual requirements of an experimental setup.

In contrast, commercially available devices are very expensive and as closed soft- and hardware systems significantly less customizable for the user. The MyoPulser presented herein, shows a broad range of useful technical features: Extensive data recording, uniform, random and even manual pulsing, adjustable ranges for both gaps and pulses, as well as a variety of applicable pulse types.

Analysis of the MyoPulser-output confirmed sharp rectangular signals^9^, precisely following the settings applied by the user, pared with extensive data recording. Pulsing of living cells revealed remarkably exact contractions according to the applied parameters. As “interface” to the pulsed probe, graphene electrodes were used. According to Tandon et al., such electrodes^10–13^ are the ones of choice due to passive biocompatibility, high availability, low price, superior charge injection characteristics as well as high resistance to chemical reactions and corrosion^14,15^.

In line with this, even after 6 h of electric stimulation, no increase of protein oxidation (protein carbonyls)^16,17^ or reduction of viability and rate of cell division was found in non-contracting HT22-cells.

This work includes detailed blueprints for versions of the MyoPulser based on different microcontrollers (“*Arduino Mega*”^18,19^ and “*ESP32*”^20^) as well as for a sample chamber. All required source codes are also included (Supplement).

In summary, the MyoPulser together with the already published analysis software “MYOCYTER” enables a low-cost but very powerful experimental setup for stimulation and analysis of any moving cell or tissue (e.g. adult and neonatal cardiomyocytes, muscle cells or beating hearts) on par with commercial solutions.

## Material and methods

### Construction and features of the “MyoPulser”

Core of the device is the electronic board “*Arduino Mega 2560 Rev3*”, programmed using the open-source “*Arduino IDE*” software (v1.8.13). The “Arduino Mega” was the board of choice, especially since it offers a large reserve of unoccupied pins (both analog and digital) for additional/individual hardware expansions. The alternative (“digital”) version of the MyoPulser, also presented herein, uses an “*ESP32*”-microcontroller-board, but depends, in contrast to the “analog” (Arduino-based) version, on connection to a computer running additional software (the freeware “*Processing*”).

Exact construction plans for both versions, lists of all components as well as the necessary software codes can be found in the Supplement. The construction of the Arduino-based MyoPulser is modular, different functions are grouped in blocks that can be switched on and off, the assembled device is depicted in Fig. S1 (Supplement). One focus was that all functions are directly adjustable via the according hardware. Manual pulsing can even be operated blindly without taking eyes off the sample during microscopy.

The following technical parameters refer to the Arduino-based version of the MyoPulser. Switching between the three different “pulsing modes” available is done via toggle switches: “Normal-mode” applies precise pulses (time of actual electrical stimulation) and gaps (unstimulated time between two subsequent pulses) in a user defined length (pulses from 1 to 100 ms, gaps from 24 to 2000 ms, a range equal to 0.5–40 Hz). In “Random-mode”, both pulses and gaps are applied randomly in a user defined range (pulses between 1 and 200 ms, gaps from 34 to 2000 ms, about 0.45–29 Hz). “Manual-mode” enables application of single pulses of defined length (1–200 ms), triggered via a push button. If necessary, the mentioned ranges can also be changed as desired via the control software.

The minimal gap times (24 and 34 ms, respectively) are due to the limited computing speed of the Arduino. This issue is described in detail in the section “Precision of time measurement for pulse and gap” of the “Results”.

Please note that the random numbers generated for “Random-mode” by an Arduino using the software-command “random()” in the controlling code of the device are repeating sequences. Here, the additional command “randomSeed()” can be applied to initialize the random number generator with random input generated using the command “digitalRead()” on an unconnected digital pin. Thus, the random sequences that are applied to samples are NOT reproducible. If reproducible sequences are still required, the code can be customized accordingly by setting a user-defined fixed seed.

**Figure 1:**
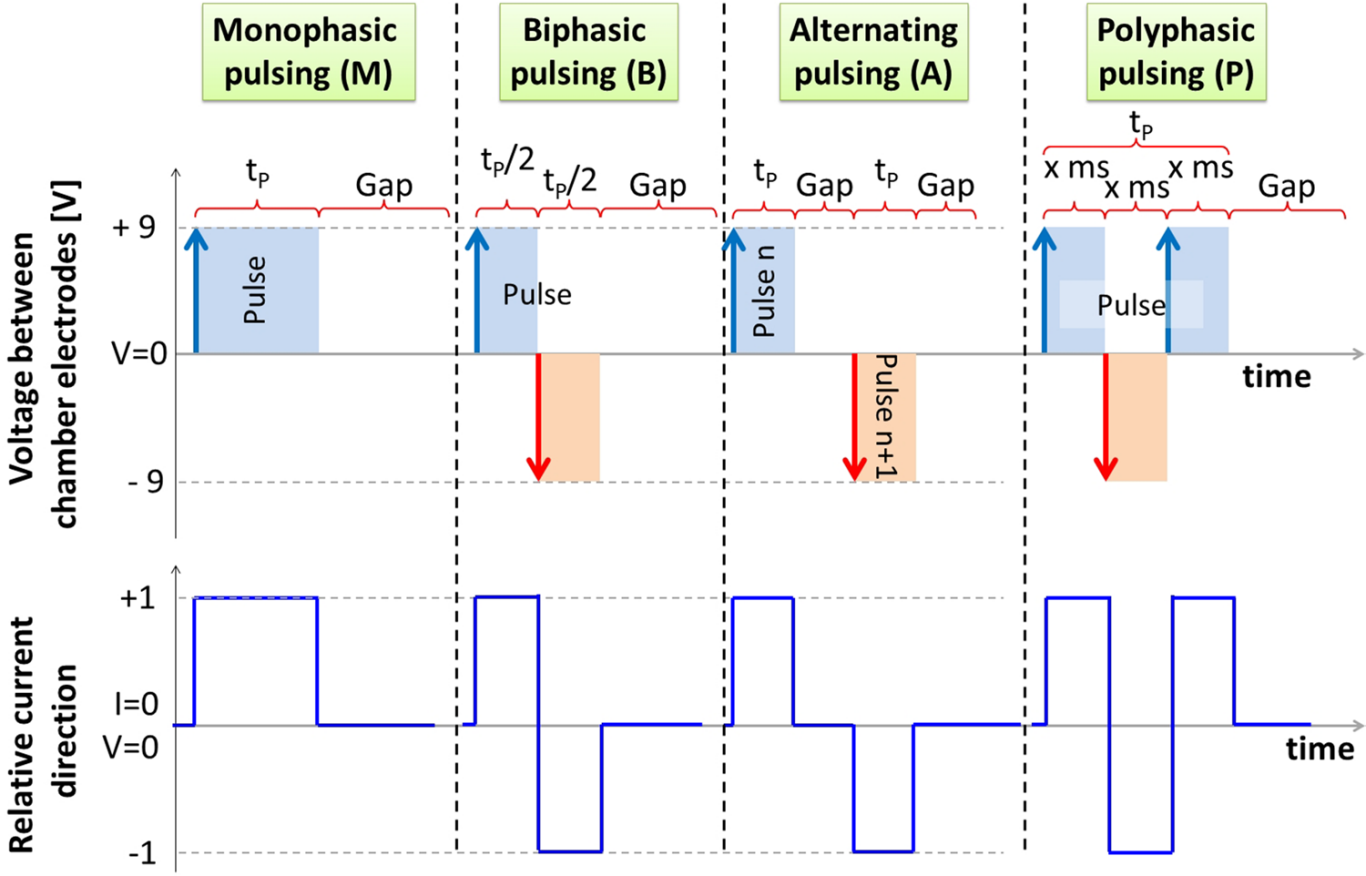
Pulse types of the MyoPulser-device. The upper part of this figures displays the four different pulse-types available by the MyoPulser: Monophasic (M), Biphasic (B), Alternating (A) and polyphasic (P). Polarization or its inversion is indicated by vertical arrows. Pulse length (t_p_) is depicted as color-filled area, gap length (Gap) without electrical stimulation are just white. Polyphasic pulsing is characterized by a rapid change of polarity, adjustable in the range of milliseconds (“x ms”). The part below depicts the according relative direction of the electric current, following the potentials’ direction between the electrodes of the sample chamber as shown above.

Besides of the three pulsing modes, also four different “pulse types” (depicted in Fig. 1) that change the characteristics of the output signal can be applied: “monophasic”, “biphasic”, “alternating” and “polyphasic”.

“Monophasic” pulses do not change their polarity, “biphasic” ones invert polarity after 50% of the pulse length, realized via a “motor controller”-board (dual H-Bridge motor driver, L298N-module), able to change polarity of the chamber within 10 µs.

“Alternating” pulses invert polarity from pulse to pulse applied.

“Polyphasic” pulses change polarity several times during the current pulse, basically applying high-frequency alternating current.

The frequency of polarity changing can also be adjusted by the so-called “phase-multiplicator”, ranging from below milliseconds up to 25% of the overall pulse length. For instance, if a pulse length of 10 ms is applied and the “phase-multiplicator” is set to 1, the polarization changes every single millisecond. If the “phase-multiplicator” is set to 2, the polarization changes every two milliseconds. If it is set to zero, polarization is inverted as fast as the microcontroller is capable of (repolarization at about 32 kHz). However, the repolarization time must not exceed 25% of the total pulse duration, otherwise biphasic pulsing should be applied. It is also possible to set an exact duration in microseconds for each repolarization.

During a pulse, a voltage (herein 9 V) is applied between the electrodes located in the sample, with one of the electrodes serving as the positive (anode) and one as negative (cathode); and electrodes become polarized. Subsequently, both Faradaic and non-Faradaic reactions^21^ can occur; the latter including a redistribution of charged particles in aqueous solution. In contrast, Faradaic reactions actually transfer electrons between electrode and electrolyte in aqueous medium covering the sample, resulting in reduction or oxidation of chemical species. Both reactions can be either reversible or irreversible. During reversible reactions chemical species remain bound or close to the surface of the electrode.

Irreversible reactions, e.g. reduction of water, can release hydroxyl ions, increasing pH of the environment, and gaseous hydrogen, moving away from the electrode. The same applies to the oxidation of water, which releases protons, reducing pH of the environment, and gaseous oxygen. Thus, irreversible Faradic reactions can change pH of medium over time during the experiment, potentially forming species that may damage both sample and electrodes.

During a first monophasic pulse, the cathode charges and the injected charge is stored reversibly in the double layer capacitance between the surface of the electrode and the surrounding aqueous medium, while the potential becomes more negative. Further charge injection (due to a longer pulse time) results in Faradaic currents, electron transfer and Faradaic reactions.

The second monophasic pulse will already be applied to a cathode with a more negative potential than the first one. Thus, a smaller fraction of the injected charge will be stored reversibly in the double layer capacitance, resulting in an earlier onset of Faradaic currents and irreversible formation of potentially toxic products. With ongoing monophasic pulsing, Faradaic currents become maximized.

That effect can be significantly reduced by application of biphasic or polyphasic pulses (see above). Applying those, the first phase (stimulating phase) triggers the biological effect like contraction of a cardiomyocyte, while the second phase (reversal phase with inverted polarization) reverses the chemical processes induced by the first one. This minimizes Faradaic currents and also irreversible formation of toxic by-products. These briefly presented effects were described in very detail by Merrill et al.^22^.

Furthermore, Laasmaa et al.^9^ were able to show that application of multiple pulses in a short sequence led to a reduction of the threshold voltage required for stimulation when compared to a single pulse, also reducing the probability of Faradaic reactions.

Thus, switching electric polarity during the pulse as well as application of short pulse times (≤ 10 ms) significantly reduces formation of potentially (cyto)toxic electrolysis products within the sample medium. Application of biphasic or polyphasic pulses also counteracts some of the irreversible reactions that electrodes produce at the interface with culture medium. Furthermore, this significantly reduces artifacts that arise during measurement of cellular action potentials due to electrical stimulation of the cells via pulsing^23^.

Important data (pulse and gap lengths, ranges of both pulse and gap length in “Random-mode”, experimental time, overall pulses as well as applied pulse type and phase-multiplicator) are shown in real-time on a liquid crystal display (LCD) (see Fig. S8, Supplement). Detailed experimental data (applied settings and ranges, switching between different pulse modes, single and cumulative times for gaps and pulses, applied overall pulses, as well as phase-multiplicator) are also sent via USB to a connected computer and can be read out using the Arduino IDE (using the “Serial Monitor”).

Since both Arduino and ESP32 only supply a maximum voltage of 5 V, pulses are powered by a switchable 9 Volt block battery, that can be tested via a connected digital voltmeter.

For the first start-up of the Arduino-based MyoPulser after assembly or a calibration of the device (maybe for use of it in exceptional ambient temperatures) a function for hardware diagnostics was also integrated into the software. Since the ESP32-based version functions without analog components, an initial calibration is not necessary.

### MyoPulser control software

Codes (included in the Supplement) of the MyoPulser are written in a “C/C++”-based language and have to be uploaded to the main electronic boards “Arduino Mega 2560 Rev3” or “ESP32”, respectively, via the free “*Arduino IDE*” software (v.1.8.13).

For proper function of the ESP32-version, the free software “*Processing*” (v.4.0 beta 6) is also necessary. The according code (written in “Java”) is also found in the Supplement.

### Construction of the cell chamber

The cell chamber is depicted in Fig. S10 (Supplement) and described in detail in the Supplement. The electrodes stimulating the medium-covered sample are made of carbon/graphene (pencil lead)^13^. Metal electrodes (copper, zinc or their alloys) will become highly toxic for sample cells through electrolysis within minutes, while the used graphite is virtually inert.

During the experiments, the distance between the electrodes in the chamber was 5 mm; consequently, the 9 V potential difference applied using a block battery induce an electric field strength of 1800 V/m. Depending on the pulsed cell/tissue type, the distance between the electrodes may be changed^13^.

### Microscopy and video recording

Samples were recorded at 20-fold magnification in transmission light mode (Olympus IX53) at 240 frames per second (fps) for 30 s, using a middle class smartphone (Xiaomi “*Mi Note 10 Pro*”) attached to the ocular of the microscope via an adapter (Vizzlema “*Universal*”)^1^.

For evaluation via the macro “MYOCYTER”, videos were converted from “mpeg”-format into an “*ImageJ*”-compatible “avi”-format (mjpeg-compression) without any visible loss of quality using the software “*Any Video Converter*” (v.6.3).

### Mice, cardiomyocyte isolation, treatment and experimental setup

Male C57BL/6J (“black 6”) mice (20 weeks old; Charles River Laboratories) were housed at 22 °C with a 12 h light–dark cycle (lights on at 6.00 a.m.) in type II Makrolon cages. Mice were exclusively scarified to collect organs and blood for scientific purposes, no further approval by national ethics committee was needed (§7 Abs. 2 TierSchG). Animals were anesthetized (isoflurane 3–5%) prior to cervical dislocation, heart was removed and cardiomyocytes were isolated according to an isolation protocol modified after Ackers-Johnson et al.^24^. Isolated cardiomyocytes were kept and also pulsed in a Krebs–Ringer bicarbonate buffer (137 mM NaCl, 5.4 mM KCl, 0.5 mM MgSO_4_-heptahydrate, 10 mM d-glucose, 1 mM CaCl_2_-dihydrate, K_2_HPO_4_-trihydrate, 25 mM NaHCO_3_).

After a short settling time (30 s), cells were electrically stimulated (pulsing frequencies from 1–2.5 Hz, bi-phasic pulses of 9–40 ms, 9 V, with an electric field strength of 1800 V/m) via MyoPulser in the cell chamber described above.

### HT22 cell culture

HT22 cells (mouse hippocampal neurons) were cultured at 37 °C and 5% CO_2_ in uncoated T75 Flasks (ThermoFisher Scientific, #156499) or poly-L-lysine-coated glass bottom petri dishes (MatTek, #P35GC-1.0-14-C) for fluorescence microscopy. As medium high glucose DMEM (“HyClone Dulbecco's Modified Eagle Medium (DMEM) with high glucose”, Cytiva, SH30243.01), containing 10% heat-inactivated fetal bovine calf serum (FBS), 1% penicillin/streptomycin, 1% GlutaMin, and additional 0.35% glucose was used.

### Pulsing of HT22 cells

For proper attachment, cells were plated on glass bottom dishes 24 h before the experiment. One control was kept in the incubator under growth conditions. A second control (“unpulsed”) was sham exposed at room temperature (unconnected graphene electrodes were immersed into the medium), while the electrodes in the “pulsed” sample were actually connected to the MyoPulser. Cells were electrically stimulated for 6 h (6 ms biphasic pulses, gaps of 1 s, more than 21,000 pulses applied).

### Immunocytochemistry of protein carbonyls

Protein carbonyls were immunostained using a modified protocol according to Ref.^16^.

The primary antibody (Sigma-Aldrich, D9656-2ML) was applied in a dilution of 1:200, as secondary one AlexaFluor 633 (ThermoFisher Scientific, A-21071, diluted 1:300) has been used, followed by embedding of the cells in “Roti®-Mount FluorCare DAPI” (Roth, HP20.1).

Samples were evaluated using a “Zeiss LSM780” confocal microscope, quantification of the images was done via a modified version of our software “Cyt/Nuc”^25^, measuring the cytosolic fluorescence intensities.

### Pulse tester

The electrical output of the MyoPulser was tested with two light-emitting diodes of clearly distinguishable colors (recommended voltage should be at about 3 V) soldered together with the respective complementary contact of the other and fit out with a 470Ω resistor (as depicted in Fig. S9, Supplement) in order to keep the maximal current below 20 mA as recommended for most diodes. Since diodes only conduct in one direction, direct current (monophasic pulse) will only light one of the two diodes (regardless of the polarity of the connection), while alternating current (biphasic or polyphasic pulses) will light them alternately according to the applied frequency. Apparent (but technically not possible) synchronous lightening of both diodes indicates high-frequency alternating current. In this simple way, polarity changes and different pulse types can be made visible directly. For precise measurement of the output signals, of course, a high-resolution oscilloscope was used.

### Voltmeter and oscilloscope

For exact testing of circuits, measuring voltage, current, frequencies and pulse-shapes, a “*Voltcraft* VC830” digital multimeter and a “*PicoScope 2104 PC Oscilloscope*” from “*pico Technology*” were used.

When using a “*PicoScope*” or any USB-connected oscilloscope, it is essential that the USB port of the Arduino/ESP32 is NOT plugged into the same computer the oscilloscope is connected to. Otherwise there may be massive disturbances of the output signal, since oscilloscope and microcontroller share the same ground (computer) during measurement.

Furthermore, the two output channels of the MyoPulser were short-circuited for the measurement with a high-impedance resistor (10 kΩ, as depicted in the Figs. S3, S11, Supplement) to simulate the connected cell chamber.

### Data extraction with “MYOCYTER”

MYOCYTER is a user friendly and peer-reviewed “*ImageJ*”/”*FIJI*”-macro^1^, written in a Java-like programming language, developed for the extraction of a large variety of parameters from high-speed videos (a minimum of 120 fps is strongly recommended) of contracting cells like (cardio)myocytes or even whole hearts, created by our group. In this publication, the most recent version (v.1.3) of this software was used.

The extracted parameters include (amongst others) the systolic (used in this work corresponding to “contraction time”) and diastolic (corresponding to “relaxation time”) share of a contraction, the overall contraction time for four different “thresholds” as well as the amplitude.

The output of this software is presented both as plot and numerical table for further statistical analysis/processing. The extracted data describe every single recognized contraction in detail, allowing very precise statistical comparisons of different samples.

To run MYOCYTER, an older version of ImageJ (1.52 s; you may also contact the corresponding author of this work) is recommended as well as a *Windows*-based operating system. *Mac OS* or *Linux* are strictly not advised, since the macro was built and optimized only on *Windows*-systems (7 and 10).

### Statistics

Numerical data of this publication were further processed and analyzed in GraphPad’s “*Prism*” software (version 8.1.2).

First, data have been tested for Gaussian distribution (using the D'Agostino & Pearson, Anderson–Darling, Shapiro–Wilk and Kolmogorov–Smirnov tests implemented in “*Prism*”). According to the result, proper statistical tests were applied. For Gaussian distributed data: one-way ANOVA (Holm–Šídák) or Student’s t-test (one-tailed, no correction), for not Gaussian data: one-tailed Student’s t-test (Mann–Whitney) or non-parametric one-way ANOVA (Kruskal–Wallis), respectively. p-values < 0.05 were considered to be statistically significant.

Applied tests and significances are indicated in the according figures.

### Updates for the MyoPulser device

Please visit either www.scyrus.de or https://github.com/Myocyter/MyoPulser-v1.0 to get the most recent versions of MYOCYTER and also updates/extensions (both hard- and software) for the MyoPulser.

### Ethics declaration

Animals were kept according the NIH guidelines for care and use of laboratory animals, also all experiments were approved by the Ethics Committee of the State Ministry of Agriculture, Nutrition and Forestry (State of Brandenburg, Germany)^26^.

### ARRIVE guidelines

This study is reported in accordance with the ARRIVE guidelines.

## Results

### Real output signals of the “MyoPulser” measured with an oscilloscope

To ensure that real signaling of the device is as close as possible to the theoretic output depicted in Fig. 1, the actual output was quantified with an oscilloscope. In order to simulate a connected sample chamber, the electrodes were short-circuited with a high-impedance resistor of 10kΩ for measurement of the real output.

**Figure 2:**
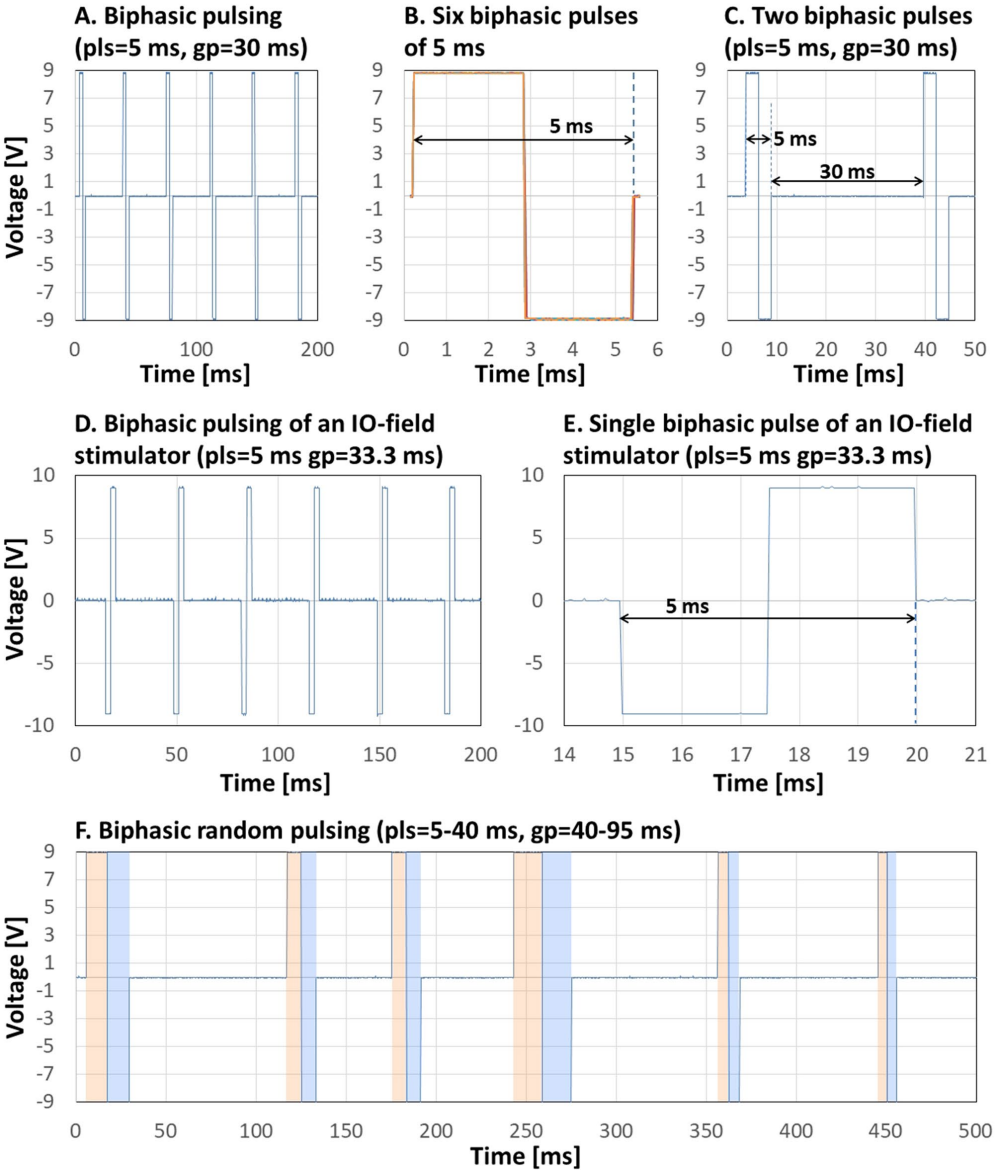
Biphasic pulses measured with an oscilloscope. (**A**) Depicts sequence of polyphasic pulses with a length of 5 ms (pls), followed by an unpulsed gap of 30 ms (gp). Depending on the polarity, the electric potential switches from + 9 to − 9 V. (**B**) Displays an overlay of the six pulses shown in (**A,C**) depicts the first two biphasic pulses shown in (**A**). (**D**) Shows a biphasic pulse sequence (5 ms pulses followed by a gap of 33.3 ms) from a commercial device (“MyoPacer” from *IonOptix*), (**E**) a magnified representation of a single biphasic pulse (5 ms) of the mentioned device. (**F**) Depicts a sequence of biphasic random pulses, generated by MyoPulsers’ “Randomizer”-function. The single pulses range from 5 to 40 ms, followed by a unpusled gap ranging from 40 to 95 ms. For better visualization, the two phases of every pulse are indicated by different colors.

Biphasic pulsing switches chamber polarity after 50% of the applied pulse time. As shown in Fig. 2B, both phases of the pulse were of the exact length, also overall pulse length exactly followed the applied settings. Shape of the pulses was highly replicable (Fig. 2A–C). Pulses are square waves with very high slope and low noise, the beginning pulse rises from 0 to 9 V within 4.5 microseconds, polarity is inverted in about 10 microseconds (Fig. 2B). The biphasic output of the MyoPulser can also be compared to the one of a commercial device (IonOptix’ “*MyoPacer*”) (Fig. 2D,E).

In random mode, biphasic pulses follow the pulse and gap ranges as defined by the user (Fig. 2F).

**Figure 3:**
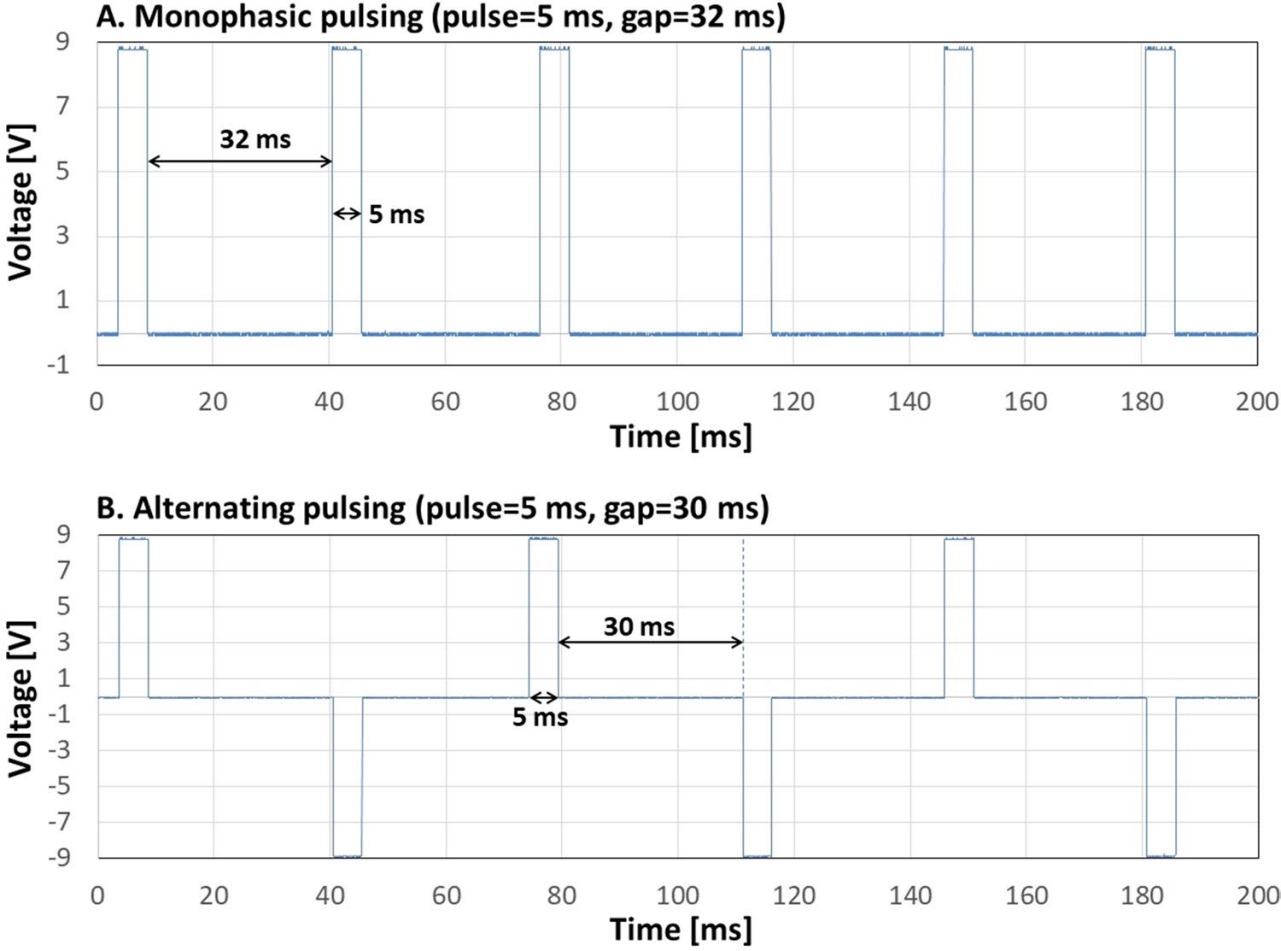
Monophasic and alternating pulses measured with an oscilloscope. (**A**) Shows a sequence of six monophasic 5 ms-pulses, followed by a gap of 32 ms. (**B**) Shows a sequence of six alternating 5 ms-pulses, followed by gaps of 30 ms.

Monophasic pulsing also revealed high precision following the user-applied settings, as well as high reproducibility of the signal (Fig. 3A), as well as alternating pulsing (Fig. 3B).

**Figure 4:**
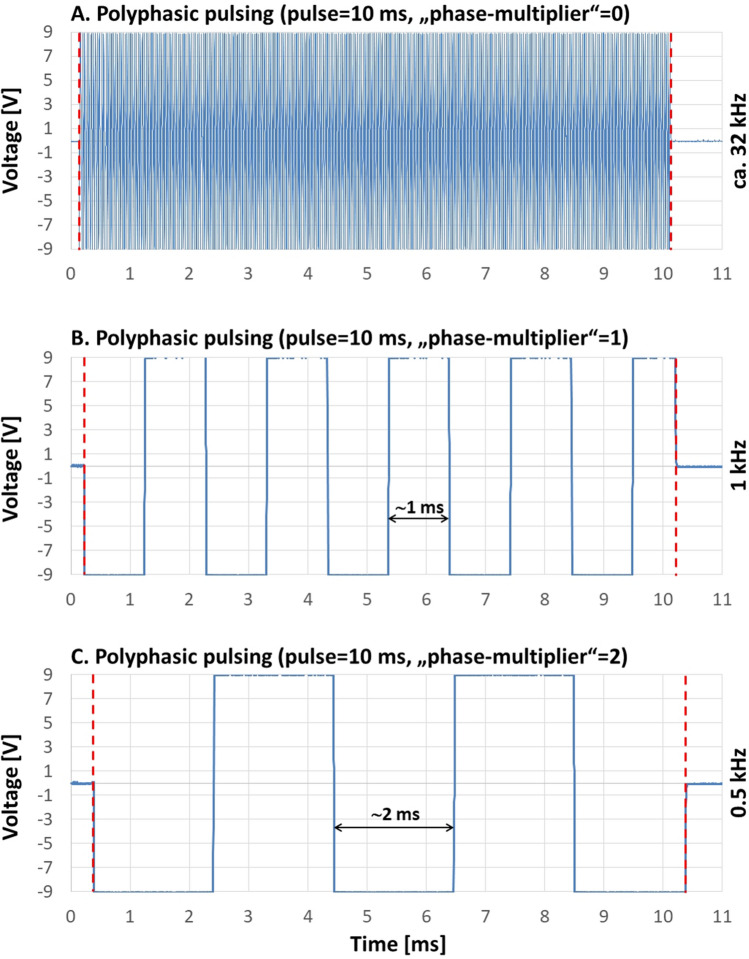
Multiphasic pulsing and effect of the “phase-multiplier”, measured with an oscilloscope. (**A**) Shows the measured output of the MyoPulser, with “polyphasic”-pulsing, a pulse time of 10 ms and a “phase-multiplier” of P = 0 applied. The AC-frequency of the according pulse was about 32 kHz, the maximum frequency the device can deliver. Increase of the phase-multiplier to P = 1 resulted in polarity inversion every single millisecond (1 kHz), shown at (**B**). Further increase of the multiplier to P = 2 results in polarity inversion every 2 ms (0.5 kHz), displayed in (**C**). The vertical dashed lines include the adjusted pulse time of 10 ms.

Polyphasic pulsing is virtually application of high frequency alternating current (AC) to the sample chamber. As depicted in Fig. 4, the “phase-multiplier” defines the AC frequency of the pulse following the function multiplicator-1 kHz. Though, in this case a multiplier of zero returns the highest AC frequency of which the device is capable of: about 32 kHz (Fig. 4A). An increase in the multiplier leads to a corresponding decrease in the AC frequency (Fig. 4B,C). As shown in Fig. 4B, the last phase of a 10 ms-pulse, changing its polarity every millisecond, is slightly shortened in order to maintain precisely the user specified pulse length. The next section explains the technical background of this behavior in detail.

### Precision of time measurement for pulse and gap (Arduino-based version of the MyoPulser)

Due to the limited computing speed of the Arduino (ATmega2560 microprocessor), duration for cycling the control program takes between 24 and 34 ms in a mode-dependent manner. The time necessary for the device to process and calculate all of the required data between triggering of two subsequent pulses is a “forced gap”, that cannot be undercut. This issue has been solved by adding code that determines the exact cycle time of each single pulse using the internal clock of the Arduino. The “forced gap” is correctly offset against the user determined one, which is usually well above 300 ms using cardiomyocytes. Nevertheless, a small deviation between the overall experimental time and the sum of all gaps and pulses still remains.

In “normal-mode” (1000 ms gap, 5 ms pulse, polyphasic pulsing with a phase-multiplicator of 0, while 9501 pulses were applied), the device displayed an overall running time of 9561.32 s, a gap-sum of 9503.997 s and a pulse-sum of 47.505 s. Thus, the sum of all gaps and pulses is only about 99.9% of the overall experimental time. The forced gap for all pulses was 24.42 ± 0.62 ms, the absolute gap was 1000.42 ± 0.7 ms, while the pulse of 5 ms had no standard deviation.

In “random-mode” (gaps between 34 and 2000 ms, pulses between 1 and 200 ms, polyphasic pulsing with a phase multiplicator of 0 and 4852 applied pulses), overall experimental time was 5451.111 s, sum of gaps was 4962.036 and sum of pulses 484.78 s. Here, both added up 99.92% of overall time. Average pulse time was 99.91 ± 57.55 ms, average gap time 1022.68 ± 570.37 ms and forced gap 34.59 ± 0.64 ms.

Those slight differences seem to be the result of rounding errors, since the smallest used time step is one millisecond. As expected, the deviations are therefore in the range of ≤ 0.1% s.

The “forced gap”, which is a minimum of 24 or 34 ms respectively, does not restrict usability of the MYOPULSER, especially as cardiomyocytes pulsed with gaps below 200 ms virtually just stop contracting according to our experience. Using those cells, typically applied gaps are found in a range from 500 to 1000 ms, a multiple above the “forced gap”.

To execute lines of code necessary to invert chamber polarization with a phase multiplicator ≥ 1, the microprocessor takes about 21.4 µs. This slight deviation accumulates with every single repolarization. As can be seen in Fig. 4B, the last phase of the pulse is slightly shorter than 1 ms. This results from the software solution of this problem: The accumulated deviation is subtracted from the last phase in order to maintain exactly the pulse length as defined by the user. In this case, ten repolarizations accumulate an error of about 0.214 ms, trimming the last phase to about 0.786 ms, while the pulse ends exactly after the preset length of 10 ms.

Code optimizations kept the pulse time error for all pulse types and lengths due to rounding and limited computing speed of the controller in a very low range from 0.5 to 0.05% of deviation (measured with the oscilloscope) from the actual applied settings.

Also, maximum AC frequency of 32 kHz using polyphasic pulsing *during* a pulse is not in contradiction to the above described “forced gap” of at least 24 ms *between* two subsequent pulses.

The forced pause (“forced gap”) between two consecutive pulses of 24 ms is due to the limited computing power of the used microcontroller. Several hundred lines of code must be processed from pulse *n* to pulse *n* + *1*, results are calculated and sent to the COM port which connects the device with a computer.

In contrast, reversing the polarity *during* the current (polyphasic) pulse applied requires only two lines of code, which are processed over and over again until the pulse is finished. This is of course less computationally intensive and thus much faster. Thus, it is possible to apply polyphasic pulses (that change polarity several times) with a user adjustable repolarization frequency of up to 32 kHz, even despite the low computing power of the “*Arduino*” (see Fig. 4).

Though, even when using a significantly faster microcontroller than the “*Arduino*”, the technical limitations of the used motor control, which requires a switching time of 10 microseconds, would limit the AC frequency of a polyphasic pulse to a maximum of 100 kHz.

### Cytotoxicity of electrodes in vitro

Classic metal electrodes containing large amounts of copper, iron, aluminum, nickel, tin, chrome or zinc segregate with every pulse highly toxic electrolysis products into the medium covering the sample, resulting within minutes in extreme (oxidative) stress or even cell death, influencing the experimental outcome (data not shown). To avoid this, pencil graphite electrodes (PGE) were used that turned out to be biologically inert and did not reveal any cytotoxic effects even after several hours of pulsing.

Electrical stimulation of HT22 cells (6 h, more than 21,000 pulses applied, 6 ms pulse time (both bi- and poly-phasic), 1000 ms gap, at room temperature in culture medium) did not result in an increase of dead cells or a decreased viability compared to an unpulsed control with and without electrodes contacting the medium. Also, no cell fragments were found floating in the medium after treatment (data not shown).

**Figure 5:**
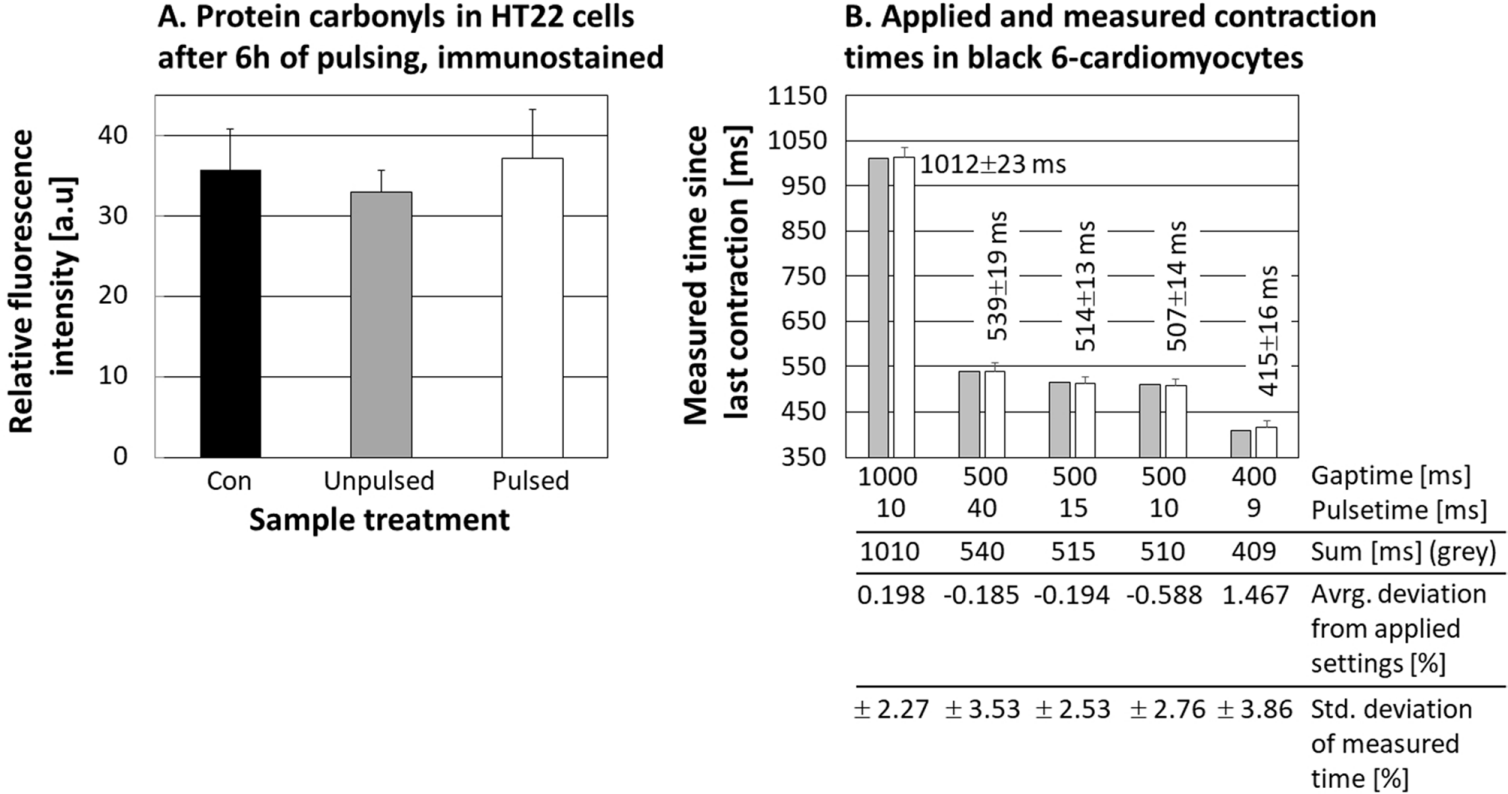
Biological application of the MyoPulser. (**A**) Shows the amount of protein carbonyls induced in cells by pulsing the sample for 6 h (biphasic pulses of 6 ms, gaps of 1 s). “Con” indicates a control kept for 6 h in the incubator under growth conditions (black column), the second control (“unpulsed”) was sham exposed (electrodes contacted the medium, but no pulsing was applied) besides of the “pulsed” sample (grey), while “pulsed” represents the sample actually electrically stimulated (white). No statistically significant differences were found between the samples (neither via ANOVA (Holm-Šídák) nor Student’s t-test, one-tailed, no correction, p < 0.05). (**B**) Compares the settings applied to the MyoPulser and the actual contraction times of pulsed cells, quantified using the software MYOCYTER. The grey columns indicate the sum of pulse and gap lengths actually applied using the MyoPulser, compared to the according averaged times between two subsequent contractions of the pulsed sample cells (white columns) in ms. “Average deviation from applied settings” (table below the panel) indicates the difference between applied settings and measured average times between two subsequent contractions in percent. “Standard deviation of measured time” indicates deviation from the actual measured cellular averages in percent (for example, 23 ms are 2.27% of 1012 ms).

As marker of oxidative stress, formation of protein carbonyls, the most common oxidative modification^16,27^, was used. Though, no statistically significant difference between incubator control (cells kept under growth conditions for 6 h, unpulsed), sham-exposed (outside of the incubator, electrodes contacting the cell medium, but no pulsing applied) and actually pulsed samples (outside of the incubator, electrodes contacting medium, electric stimulation applied) was found, as depicted in Fig. 5A.

It has to be also considered that 6 h are about 10–12 times longer than an experiment with freshly isolated cardiomyocytes usually takes.

### Functionality of the MyoPulser in vitro

To test functionality of the device on a biological sample, isolated B6 cardiomyocytes, a representative and widespread commonly used cell model contracting by electrostimulation and very established in our laboratory, were stimulated via pulses ranging from 9 to 40 ms interrupted by gaps of 400–1000 ms, as commonly applied in experiments with such cells.

The according results are depicted in Fig. 5B.

The software MYOCYTER^1^ was used to extract the necessary parameters from high speed videos of the contracting cells. The averaged times between subsequent cellular contractions were found to be very close to the sum of the according pulse and gap times applied. Averaged deviations of the actual cellular contraction times from the settings applied via the MyoPulser were found in a range between − 0.194 and + 1.467%.

### The ESP32-based version of the MyoPulser

In contrast to the “analog” version (settings are applied/changed using analog hardware components like potentiometers) described above, running on an “*Arduino Mega 2560”*, also a “digital” version of MyoPulser has been constructed using an ESP32-microcontroller (*Espressifs “ESP-32S ESP-WROOM-32”*-board), where settings are applied via the user interface of a software running on a connected computer.

Both devices are using the same hardware components connected to the sample-chamber (Dual H-Bridge motor driver, L298N-module), thus, the ESP32-based version was not tested in a biological application, but only examined with regard to its output via an oscilloscope.

While the Arduino-based version applies also “intermediate steps” to the sample, if new settings are adjusted via potentiometers, the ESP32-based one applies new settings immediately and stepless after a mouse click.

Data recording is as detailed as that of the analog version, but here, both the “system time” (internal computer clock, adjusted based on “Coordinated Universal Time”, UTC) of the connected computer and the internal clock of the ESP32 are used to record various events. For example, application of new settings uses the system time of the computer, while exact pulse and gap duration during random pulsing are recorded using the timer of the ESP32. The resulting difference after 6 h of pulsing (all modes and all pulse types applied) between system time of the connected computer and internal clock of the ESP32 was 882 ms (0.0049%). Another computer (same processor, comparable hardware) with another ESP32 connected, produced a difference of 384 ms after 3 h of pulsing (only “Normal-mode” applied), that equals 0.0036%.

The ESP32-based version covers a range of 1–100 ms for pulse and 20–2000 ms for gap in normal mode, 1–200 ms pulse (manual pulsing), as well as 1–200 ms pulse and 20–2000 ms gap time (random mode). Repolarization time of the AC in polyphasic pulsing can be set in a range from 30 to 1000 microseconds in steps of a microsecond. Available modes and pulsing types do not differ, all data collected during the experiment are written immediately into a log file on the hard disk of the connected computer.

**Figure 6:**
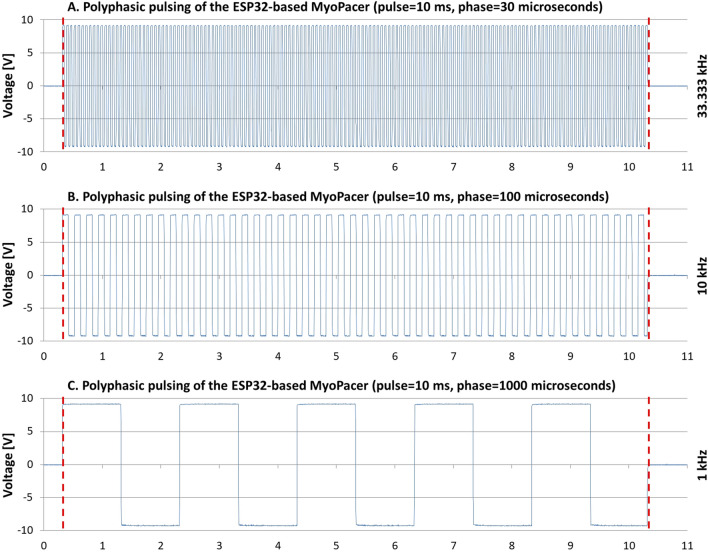
Polyphasic pulsing of the ESP32-based MyoPulser. This figure depicts a 10 ms polyphasic pulse (marked by vertical dashed lines) with different phase constants applied. Here, “phase” represents the repolarization time in microseconds (30 in (**A**), 100 in (**B**) and 1000 in (**C**)). On the right side of each panel, the according alternating current frequency is given.

Figure 6 depicts the output of the ESP32-based MyoPulser as measured with an oscilloscope, while increasing “phases” are applied, that define the frequency of the alternating current during polyphasic pulsing.

Also here, high frequent alternating current up to 33.33 kHz (phase = 30 ms) with clear rectangular signals is produced (Fig. 6A).

Due to the superior computing speed of the ESP32 compared to the Arduino and outsourcing of quite a few operations to the connected computer, the last repolarization of a polyphasic pulse is only insignificantly shortened, to maintain precise pulse length as set by the user (visible in Fig. 6B, phase = 100 microseconds). This issue is discussed in the “Precision of time measurement for pulse and gap” section.

At phase = 1000 ms, no shortening of the last repolarization is visible (Fig. 6C).

In contrast to the “*Arduino*”-based MyoPulser, the forced gap of the ESP32-based version is not caused by limited computing power of the microprocessor. Though, the minimal applicable gap here is set to 20 ms, resulting from the frame rate of the software “*Processing*” which has a default setting of 60 Hz (equals about 17 ms), a frame rate that virtually every computer of the last decade is capable of. If gaps of less than 20 ms are required, the frame rate can be adjusted in the software, but requires a corresponding processing power of the connected computer. If that computer is not able to supply the necessary frame rate, the deviation between computer clock and system clock of the ESP32 may sum up several seconds.

## Discussion

MyoPulser turned out to be a relatively simple to build and powerful device that delivers functions like polyphasic pulsing with definable frequency and extensive data recording, not yet available even with corresponding commercial devices.

Settings are applied to the sample with high temporal precision and can be changed quickly and accurately via a user-friendly interface. MyoPulser produces clear rectangular signals, the used pencil graphite electrodes are biologically inert.

As far as we know, the MyoPulser is the first non-commercial do it yourself field stimulator presented in a peer-reviewed publication.

Given the possibility to apply also high frequency alternating current, a technical feature that is not available for example in the commercial device of the manufacturer IonOptix (see Table 1), our presented device can also be used in neuroscience to trigger action potentials with only a few adaptations of the software. The MyoPulser thus has application potential beyond myophysiology and can be used together with the open source analysis tool MYOCYTER.

**Table 1:** Features of the ESP32- and Arduino-based MyoPulser compared.

Feature	ESP32-based MyoPulser	Arduino-based MyoPulser
Adjustment of the settings	Digital via sliders of a software; no intermediate values when settings are changed	Manual via rotary potentiometers; intermediate values are possible when settings are changed during an experiment
Connection to a computer for proper function via USB	Mandatory	Optional
User interface without submenus	Via software	Via hardware
Output signals comparable with commercial devices	Yes (compared to IonOptix’ “MyoPacer”)	Yes (compared to IonOptix’ “MyoPacer”)
Data recording for each individual pulse with all settings applied	Automatically written to a file	Manually via copy/paste
Estimated production costs [$]	55	100

Feature	MyoPulser (both versions)	IonOptix’ “MyoPacer”
Polyphasic pulsing	Yes	No
Quick change of settings without submenus	Yes	Submenus, only accessible via a single rotary decoder
Pulsetypes	Monophasic, biphasic, alternating, and polyphasic	Monophasic, biphasic, and alternating
External trigger	Yes (instead of manual pulsing)	Yes
Pulse length [ms]	1–200 (depending on pulse-mode)	0.4–90
Voltage [V]	9; adjustable, if a laboratory power supply is used	0–40
Extensive data recording for every single pulse	Yes	No
Pricing [$]	55–100	About 3000
Electrode material	Carbon/graphene (cheap, disposable)	Platinum (expensive, not disposable)

Thus, the open source code of the “MYOCYTER”, the low cost of the MyoPulser as well as the ease of use and customizability of both reduce the entry threshold into the field of cardio(myo)physiology considerably and can thus give new impulses to the research field.

All in all, the goal of adding a suitable powerful and complementary hardware in form of the MyoPulser to our software “MYOCYTER” was achieved.

### Voltage limit of the 9 V-block battery

In our experiments, a 9 V-block battery was used to provide electrical stimulation. If, for any reason, this voltage is not sufficient, the block-battery can be replaced by a laboratory power supply. In this case, the motor-control has to be replaced also, since the used board (“L298N-module”) is limited to a maximum of 10 V. Please also consider that a “fresh” 9 V-block battery may provide up to 9.6 V and can decrease over time to even about 5 V.

### ESP32- and Arduino-based MyoPulsers compared to a commercial device

Choice of the microcontroller depends on the user’s needs. Both versions differ from each other, but are still comparable to a commercial device (*IonOptix*’ “MyoPacer”), as displayed in Table 1.

## Outlook

The next logical step is the real-time connection of pulsing, detailed data recording and evaluation of cellular contractions into a single data stream.

To achieve this, initial experiments have already been carried out with a microscope that allows light from a laser diode to pass through the sample under investigation, and onto a light-sensitive array of extremely fast responding photodiodes or phototransistors, respectively. Such an array converts changes in illumination (effectively what the MYOCYTER software does) into a changing voltage correlated with the according cellular contractions at a frame rate of about 1500 per second.

## Supplementary Information

Supplementary Information 1.

Supplementary Figure S1.

Supplementary Figure S2.

Supplementary Figure S3.

Supplementary Figure S4.

Supplementary Figure S5.

Supplementary Figure S6.

Supplementary Figure S7.

Supplementary Figure S8.

Supplementary Figure S9.

Supplementary Figure S10.

Supplementary Figure S11.

Supplementary Figure S12.

Supplementary Figure S13.

Supplementary Figure S14.

Supplementary Figure S15.

Supplementary Figure S16.

Supplementary Figure S17.

Supplementary Figure S18.

Supplementary Information 2.

Supplementary Information 3.

Supplementary Information 4.

## Data Availability

Datasets generated and/or analyzed during the current study are available in the Supplement of this publication. The confocal microscopy data for the measurement of protein carbonyls (depicted in Fig. 5) can be found in the Supplement (“Images CLSM protein carbonyls in HT22 cells.zip”), including the ImageJ macro for image analysis. The oscilloscope measurements of the device (Figs. 1, 2, 3, 4, 6) in the file “Output signals (oscilloscope).zip”. Those files contain all of the raw data used for this publication.
