# Supplementary material for: The MyoPulser field stimulator, a do it yourself programmable electronic pacemaker for contracting cells and tissues
Source: Sci Rep. 2023 Feb 11;13:2461. doi: 10.1038/s41598-023-29145-3 (PMC9922332; doi:10.1038/s41598-023-29145-3)
Supplement: Supplementary file 1 — Supplementary Information 1. [file 41598_2023_29145_MOESM1_ESM.docx]

**The MyoPulser field stimulator,**

**a do it yourself programmable electronic pacemaker for contracting cells and tissues**

Christiane Ott^1,2^ and Tobias Jung^1,2^

1 Department of Molecular Toxicology, German Institute of Human Nutrition Potsdam-Rehbruecke
 (DIfE), 14558, Nuthetal, Germany.

2 German Center for Cardiovascular Research (DZHK), 10117, Berlin, Germany.

**Corresponding author**: Tobias Jung ([tobias.jung@dife.de](mailto:tobias.jung@dife.de)), ORCID: 0000-0002-9159-8444

**Supplement**

**MyoPulser overview and technical notes**

*IMPORTANT*: Please read the *entire* supplement of this publication before starting construction of the device. The first part covers the “analog” (Arduino-based), the second one the “digital” (ESP32-based) version of the MyoPulser.

As depicted in **Fig. S1**, the MyoPulser is divided into the functional units “Basic Box”, “Randomizer Box” and “Definable Box”. The according construction plans and necessary connections of the hardware are depicted in **Fig. S2-S5**.

For someone with slight experience, assembly of the device is possible in about one day.

**Installation of the “Arduino IDE”**

The software “*Arduino IDE*” is a freeware available at https://www.arduino.cc/en/software.

This program is downloadable as ZIP-package and can be used right away after extraction without any installation or administrator rights. This IDE is necessary to compile and load code to a microcontroller like the used “Arduino Mega”, which can then perform the necessary functions as the central control unit of the MyoPulser.

Though, for the ESP32-based version, the “Arduino IDE” has to be prepared via additional steps:

“Additional Boards Manager URLs” have to be added under “File > Preferences” (in the Arduino IDE). The two following links have to be added, separated via a comma:

https://dl.espressif.com/dl/package_esp32_index.json, http://arduino.esp8266.com/stable/package_esp8266com_index.json

Then you have to install “libraries” that allow the IDE to communicate with the ESP32- microcontroller.

For this, select “Tools > Board > Boards-Manager” and install the library “ESP32” by “*Espressif Systems*”. Just type “esp32” in the headline and the software should find the according library to install it manually afterwards.

*IMPORTANT*: It is mandatory to install version 1.0.4 of this library, otherwise the device will not function properly due to communication problems between PC and ESP32-microcontroller!

Now, the Arduino IDE can be used to upload the necessary codes for both the Arduino- and ESP32-based versions of the MyoPulser; the detailed upload process for both versions is described in the according sections.

**Main components of the MyoPulser (“analog” version, Arduino-based)**

- *Arduino*® Mega 2560 Rev3 (programmable board, about $24)
- HD44780 2004 LCD Display 4x20 digits (about $8)
- Breadboard jumper wires (about $7)
- Resistors (several, 220Ω, 470Ω, 1kΩ and 10kΩ, 5$)
- Breadboard for solderless prototyping (about $5)
- Switchable plastic storage box for a 9V battery and the according battery ($5)
- Rotary potentiometers (1x B10K, 9x B5K, 5$)
- Bell wire (soft and hard) (8$)
- Toggle switches (3, 5$)
- Push buttons (2, 4$)
- LED (1, 1$)
- Alligator clips (2, 2$)
- Dual H-Bridge motor driver, L298N-module (4$)
- Voltmeter (1, 3$)
- Small cable ties (2$)
- Heat shrink tubes (2$)
- Solid plastic plate, about 210 x 300 mm (5$)
- M2 nuts and bolts of different length (10-20 mm) (5$)

Summarizing about $100. Further necessary tools are wire stripper, soldering iron and solder.

The device can be built as a “prototype” with a pluggable electronic breadbord, but it is recommended to solder parts like potentiometers, especially because otherwise loose contacts may occur, which can lead to an unintentional restart of the device and thus to data loss during an experiment. The soldered inside of the “Basic-box” is depicted in **Fig. S6** as construction proposal.

**Upload of the MyoPulser-software on the device**

After complete assembly of the device, the MyoPulser control software has to be uploaded on the board. In order to do this, the device has to be connected via USB to a computer, running the “*Arduino IDE*”.

First, set the correct board via “Tools > Board > Arduino AVR Boards > Arduino Mega or Mega 2560”.

Then, the correct USB-port has to be selected via “Tools > Port”. If connected to the computer via USB, the port with the “*Arduino*”-board should be recognized and found automatically.

The source code (also included in the supplement) has to be copied into the “*Arduino* IDE” and uploaded to the board using “Sketch > Upload”. After compiling and upload of the software, the MyoPulser can be used for the first time.

**“Hardware-Test-mode” of the MyoPulser**

After assembling the device and upload of the control software, the MyoPulser must first be calibrated. To do this, the MyoPulser has to be switched into “Hardware-Test-mode” and be connected via USB to a computer running the “Arduino IDE” with “serial monitor” activated.

The LCD-display of the device should now look like depicted in **Fig. S8** (“Hardware-Test-Mode”) and the user should see a data stream updated secondary coming via the “serial monitor” of the “Arduino IDE”, looking as depicted in **Fig. S7**.

For a better overview, only the first three columns of the data stream are shown: the timer, as well as the analog outputs of the ports “A0” and “A2”. The other analog ports (A0 to A9 are displayed) function in exactly the same way.

If the rotary potentiometers are turned now, the Arduino returns the analog signal of the according ports. Turning the potentiometer from the lower to the upper stop, the according range between 0 and 1023 should be returned.

In our example, the potentiometer connected to port “A0” covers a range between 2 (“A0Low”) and 911 (“A0High”). The potentiometer connected to port “A2” covers about the same range.

This has to be done once for every single potentiometer.

In contrast, the parts of the device, that are connected to a digital pin like switches or push buttons, have only two different states: 0 (inactive) or 1 (active).

If the analog or digital pins listed here do not change their state if the connected parts are adjusted/switched, this may indicate a connection problem or a defective solder joint. If the device is unable to switch into “Hardware-Test-Mode” (the according digital pin is not listed in the table), this may mean a problem with the corresponding switch.

After measurement of every single potentiometer, the according ranges have to be written/changed in the code of the control software right at the beginning.

To have an example, the ranges of the two potentiometers measured above have to be adjusted in the already existing code like this:

// Set the potentiometer-ranges here, change with care and HARDWARE-TESTER (see Publication for details)

// These settings depend on the physical hardware of the MYOPULSER and should be made before FIRST USE,

// ONLY ONCE after assembly, NOT before each use.

// Sets Normal Gap

int A0Low = 2;

int A0High = 911;

// Sets Normal Pulse

int A2Low = 2;

int A2High = 911;

Once this is done for ALL of the potentiometers (A0 to A9) from the output table, the device can be finally used now.

**Further testing of the MyoPulser**

A simple test of the pulsing can be done using the LED-arrangement depicted in **Fig. S9**. For a detailed analysis of the output, a professional oscilloscope should be used.

**Construction of the sample/cell chamber**

The basic structure of the cell chamber is shown in **Fig. S10**.

The distance between anode and cathode defines the electric field strength (V/m) within the chamber, while conductivity (Ω⋅m)^-1^ of the medium defines the resulting current (A). Resistance of the medium (Krebs-Ringer bicarbonate buffer) filled chamber was found in ranges from 8-10 kΩ. According to Ohm’s law (U=R⋅I), using a 9V block battery, the resulting current is found in the order of 0.9 to 1.1 milliampere. The distance between the electrodes in the chamber used for our experiments was 5 mm, which corresponds to an electric field strength of 1800 V/m. Greater distances between the electrodes (resulting in lower field strengths) are not recommended using cardiomyocytes.

If the sample chamber is filled with a suspension containing cardiomyocytes, (partial) evaporation of the medium during the experiment can occur. This may cause increasing concentration of dissolved ions, which can affect the experimental results in a time-dependent manner. In order to prevent this, a cover glass can be placed over the pencil lead electrodes, that may also directly contact the medium.

**The (digital) ESP32-based version of the MyoPulser**

Core of the “digital” version of our device is the “ESP32-S2 general-purpose development board”, based on *Espressifs* “ESP32-S2-WROVER”. In contrast to the “analog” one, that is in detail presented in this publication, the digital MyoPulser comes with a software user interface and depends on connection to a computer. The cumulative cost of this device is approximately $55, the construction plans and all of the necessary connections can be found in detail in the figures **S11-S14**. **Fig. S14** depicts a transportable soldered version of the ESP32-based MyoPulser as design suggestion.

**Main components of the “digital” MyoPulser (ESP32-based)**

- “ESP32-S2-WROVER”-board (8$)
- Resistors (5x 220Ω, 2x 10kΩ, 5$)
- Rotary potentiometer (B10K-type, 1$)
- HD44780 2004 LCD Display 4x20 digits (about $8)
- Insulated electrical wire, 0.6 mm outer diameter (about $7)
- Solderable breadboard for prototyping (11x15 cm, about $5)
- Switchable plastic storage box for a 9V battery and the according battery ($5)
- Push buttons (2, 4$)
- LEDs with different colors (3, 2$)
- Dual H-Bridge motor driver, L298N-module (4$)
- Voltmeter (1, 3$)
- Heat shrink tubes (2$)

Summarizing about 55$; additional wire stripper, soldering iron and solder are also required.

**Upload and compilation of the software necessary for the ESP32-based MyoPulser**

Both necessary software codes are included in this supplement. The first is the one that controls the ESP32. This one can be uploaded to the ESP32 in the same way using the “*Arduino IDE*” as the code for the “Arduino Mega” board. Connect the ESP32 via USP to the computer and select “Tools > Board > ESP32 Arduino > ESP32 Wrover Module” as well as the COM-port, the ESP32 is using. The other applied settings are depicted in **Fig. S15**. As mentioned, it is important to use version 1.0.4 of the “esp32”-library from “*Espressif Systems*” (as explained in the section »Installation of the “Arduino-IDE”«).

The second one provides the user interface to apply the desired settings. This code (written in Java) has to be executed using the free software “*Processing*” (4.0 beta 6). It is also possible, so export this code as standalone *.exe-file that can be executed independently of “*Processing*”.

Necessary for proper function, there are several PNG-images representing the different pulse types currently applied.

After first start of the code in “*Processing*”, a folder like this should be created (your username will be of course different): “C:\Users\jung\Documents\Processing\ESP32MyoPulser”

This folder contains the saved code (as *.pde-file) and also an additional subfolder named “data”. If this subfolder is missing, just create it. Please copy the four PNG-images (“alternating.png”, “biphasic.png”, “monophasic.png”, and “polyphasic.png”) into the “data”-folder for proper function of the software interface.

Here, while the device is pulsing, all corresponding data are written to a txt-file in the folder “C:\Users\jung\Documents\Processing\ESP32MyoPulser”, which is labelled “DD_MM_YYYY-hh-mm_MyoPulser.txt”.

In contrast to the “analog” version, the “digital” one does not need any calibration.

**User Interfaces of the ESP32-based MyoPulser**

This version has two different user interfaces. The first one is the control software that runs on the connected PC (shown in **Fig. S16**) using the software “*Processing*” and via which all settings of the device are changed and applied.

The second one is the LCD of the soldered device (see **Fig. S14**), that depicts the most important current settings (depicted in **Fig. S17**) and modes.

**Connecting problems of the ESP32-based MyoPulser via a COM-port**

Establishing this connection between ESP32 and a computer is essential for proper function of the device. A considerable amount of the necessary complex calculations and recording of the data is done by the connected computer. **Fig. S18** depicts the console of “*Processing*” after a successful connection to the ESP32.

Though, problems connecting the ESP32 to the computer may occur. In the following, the basic solution strategies are presented.

The software “*Processing*”, necessary to provide the user interface, first creates a list of all available COM-ports. In our case, as depicted in **Fig. S18**, only one port (COM-port “COM8”) was found. If no COM-ports can be found at all, the user probably needs administrator rights to install such an interface. In this case, please follow the instructions matching your operating system.

If COM-Ports are available and the according list is returned as depicted in **Fig. S18**, but the software is still NOT able to establish a connection, the user may try to change the according code executed by the software “*Processing*”.

The corresponding lines of code (found in the supplement) are marked by annotations.

In this case, the lines between the annotations

“// ********* IF AUTOMATIC RECOGNITION OF THE COM-PORT DOES NOT WORK,

// ********* THEN REPLACE THE CODE FROM HERE...”

and

“// ********* ...TO HERE JUST BY THE FOLLOWING SINGLE LINE (starting with "port = ...")...”

can be replaced by a single line of code:

port = new Serial(this, Serial.list()[0], 230400);

In this case, the zero in square brackets has to be replaced by one of the numbers from the list of COM-ports that is returned by the software (see **Fig. S18**, indicated by the red rectangle). In most cases, the first identified COM-port works ([0]), and the code can remain unchanged.

If more ports are identified, please replace the “[0]” with “[1]”or “[2]” and so on, until a free and working COM-port finally establishes connection.

**Supplement figure legends**

**Fig. S1: Overview of the assembled “analog” MyoPulser (Arduino-based)**

This figure shows the fully assembled MyoPulser and its single components (“boxes”). Arrangement of the single elements is just a suggestion and can also be implemented differently depending on the users’ requirements. The single parts are mount to a solid plastic plate. Switches enable different modes of the device, rotary potentiometers adjust the applied settings, that are displayed on an LCD (see bottom left).

**Fig. S2: Composition of the “Basic-Box” of the “analog” MyoPulser (Arduino-based)**

This figure depicts the components of the “Basic Box”. Resistors are represented by green squares, indicating the according value in Ω. Three different potentiometers are installed that regulate intensity of the display (B10K), as well as gap and pulse length (both B5K). A rotary potentiometer functions here as a voltage divider, the voltage applied to the middle pin is adjusted by turning the knob, delivering a range from about 0 to about 5 V (depending on the upstream resistor limiting the current). If this output is connected to the analog pin of the *Arduino*, the voltage range is divided into 1024 steps (10-bit resolution) and can be read out by the control software resulting in changed settings.

Switches and buttons connected to a digital pin, are fit out with a so-called “pulldown resistor” (10 kΩ), ensuring noise-free ground-potential (the signal resembles a digital “0”) until the button is actually pressed. In this case, the applied “high” voltage completely “overwrites” the high impedance pulldown resistor, resulting in a digital “1”. Without a “pulldown resistor”, even noise may already be sufficient to trigger a pulse.

At 5V, a 220 Ω resistor of the LED, that indicates every single pulse applied, connected to digital pin 10, limits the current to about 23 mA, slightly above the recommended 20 mA. Though, 220 Ω are the typical LED-resistor used in most microcontroller-based projects.

The “Monopulse-mode”-toggle switch (bottom left), pauses automatic pulsing of the sample and enables the manual pulse mode that can provide single pulses via a push button (middle left). The secondary function of this mode is interrupting automatic pulsing of the sample and thus corresponds to a “pause mode” at the same time.

Necessary holes in the plastic housing are drilled (using a wood drill at low speed, which prevents tearing of the plastic) and then filed smooth for perfect fit of the single compounds.

*IMPORTANT*: The push button for manual pulsing can also be replaced by an external electronic device which serves as trigger for the MyoPulser.

**Fig. S3: Connection of LCD-display and sample chamber to the Arduino-based MyoPulser**

The upper left part of this figure depicts proper wiring of the LCD-display to the Arduino. Display-pins are counted from left to right as shown in this panel, leaving the LCD-pins 7-10 unconnected. Display intensity can be adjusted as shown using a rotary potentiometer (on the right of the upper panel). Its 5V-pin is connected with an additional 220 Ω resistor to limit the current.

The lower panel (“Connection of the sample chamber”) depicts the “motor controller” (“Dual channel L298N DC motor driver board”) as intersection of sample chamber, 9V block battery and the Arduino. The “motor controller” is intended to control up to two electric motors, while direction of rotation of such a motor is determined by the polarity of the connections. Here, the control-board, technically a so-called “H-bridge”, is used for fast polarity inversion of the sample chamber (up to 32 kHz).

Please note that the pins “IN1” and “IN2” (connected to the Arduino) control “Motor-A” (connected to the sample chamber). “IN3” and “IN4” may be used for hardware extensions that require a second chamber, connected to “Motor-B”. The used board is connected to a 9V-block battery and its input should not exceed 10V according to the user’s manual.

The switchable voltmeter is used to check the battery voltage. New batteries may provide up to 9.6V, while in almost discharged batteries the voltage can even drop below 5V. Thus, for comparability of experiments, the voltage should be checked regularly. However, the push-button of the voltmeter has an important second function. Even if the battery is disconnected via its housing switch, the sample chamber can still be pulsed by the two capacitators (16V, 100 µF) on the “motor controller”-board. Pushing the button to activate the voltmeter, discharges those capacitators almost instantly, while the voltmeter acts as a resistor so that they are not damaged.

For instant and reliable interruption of pulsing, the device can just be switched into the manual “Monopulse-mode”.

The 10 kΩ resistor (bottom right) can replace/simulate the sample chamber for a detailed signal reading with an oscilloscope (in this case, a high impedance short circuit is made), but is NOT connected at the same time as the sample chamber.

**Fig. S4: Composition of the “Random Box” (Arduino-based MyoPulser)**

This compound applies random gaps and pulses in user defined ranges. The toggle switch (bottom left) enables the “Random-mode” of the device, while the four potentiometers are used to apply the lower and upper limits for both gap and pulse times. Also here, the potentiometers are fit out with additional 220 Ω resistors to limit current and the toggle switch is connected to ground with a 10 kΩ-“pulldown” resistor.

**Fig. S5: Composition of the “Definable Box” (Arduino-based MyoPulser)**

This box contains two potentiometers to adjust the pulse-type applied and the “phase-multiplier” in case of polyphasic pulsing. A third potentiometer is not yet assigned and can be defined arbitrarily by the user. The push button that activates both the voltmeter for battery testing and discharges the capacitators of the “motor control”-board (see **Fig. S3**) was also integrated into this box, as well as the switch for the “Hardware-test-Mode”.

“Hardware-test-Mode” is a useful function, to adjust the code of the control software after assembly of the MyoPulser-device or after replacement of compounds. In this mode, the “ranges” of the different rotary potentiometers can be measured just by turning the according knobs. For this purpose, the Arduino has to be connected via USB to a computer, running the “Arduino IDE”-software. This enables display of the measured ranges in the “serial monitor” of that software.

**Fig. S6: Technical recommendations for the construction of the Arduino-based MyoPulser)**

This figure reveals the inside of the “Basic Box”.

As far as possible, it is strongly recommended to carefully solder electrical contacts to avoid loose connections (like rotary potentiometers, switches, buttons, resistors and LEDs inside of the boxes), which can lead to a loss of measurement data, changed settings or a reset of the device. For isolation of soldered wiring, heat shrink tubes should be used, as well as small cable ties for bundling. Rotary potentiometers, buttons and switches should be labeled (embosser band) in order to avoid operating errors. Sometimes it seems strange that buttons and even rotary potentiometers are equipped with an additional resistor, but doing so, the total current flow shall be kept as low as possible, maintaining reserves for later extensions of the device.

The suggested design of the device is in such a way that the interface is spread out in front of the user and there are no submenus that would cost additional operating time.

**Fig. S7: Hardware-Test-mode of the Arduino-based MyoPulser**

This table shows the results of the “Hardware-Test-mode” while operating the rotary potentiometers (connected to the pins A0 and A2, respectively) and turning them from one stop to the other in order to show the entire range. In this case both potentiometers range from 2 to 911 (Pin A0) and 910 (Pin A2) Ohm, respectively. As seen in the column on the far right, the measured values are updated every second. The returned minima and maxima for every single of the potentiometers have to be inserted in the control code, as described (see section »*“Hardware-Test-mode” of the MyoPulser*« in the supplement).

**Fig. S8: Display and pulse-modes of the Arduino-based MyoPulser**

The display provides the most relevant information to the user in real-time. Different operating modes also have different display indications, depicted in the panels of this figure.

“*Normal-Mode*” (top left panel): “Pulse” and “Gap” times are indicated in milliseconds (“mSec”), overall “Pulses” are counted and experimental time is displayed in seconds (“Time [Sec]”). Furthermore, the used pulse-type is indicated (dashed box in every panel, “P” for “polyphasic”, “M” for ”monophasic”, “B” for ”biphasic” or “A” for “alternating”, respectively). Only in case of polyphasic pulsing, also the phase multiplier is shown (here it is “0”).

“*Random-Mode*” (top right panel): This mode applies random pulses and gaps in a user defined range. Displayed are the defined lower and upper boundaries. In this case, the gaps (“Gap”) range from 34 to 2000, the pulses (“Pls”) from 1 to 200 milliseconds (“mS”). The number of already applied random pulses is counted (“Rnd-Pulses”), as well as the time since switching the device into this mode in seconds (“Rnd-Time [Sec]”).

“*Manual-Mode*” (bottom left panel): Here, pulsing is triggered manually (“Mono-Pulser (manual)”) via a push button. „Pulse“ indicates the pulse time in milliseconds (“mSec”), „Mono-Pulses“ counts the overall manually applied pulses.

“*Hardware-Test-Mode*”: In this setting, the MyoPulser is calibrated directly after assembly, after replacement of hardware components or for use under extreme temperature conditions, which may have a significant influence on the potentiometers and resistors used. The ranges of the potentiometers and functionality of all buttons can be measured via the “Serial-Monitor” of the “Arduino IDE”-software after connecting the MyoPulser via USB to a computer running it. Another possibility to read the output of a COM-port is software like the free Telnet- and SSH-Client „*PuTTY*“. This Program is also able to write the according data into a log-file.

**Fig. S9: Display and pulse-modes of the Arduino-based MyoPulser**

If the opposite poles of two different colored LEDs are soldered (light blue circles) together (right part of the image), only one of them can light up at the same time, depending on the polarity of MyoPulsers output signal (left), generated by the motor-control. However, applying high-frequency alternating current, gives the appearance that both diodes are lit simultaneously. The resistor (470 Ω) limits the diode current to less than the recommended 20 mA (at 9V).

This setup can be used for easy and quick testing of the functionality of the MyoPulser, even during an ongoing experiment.

**Fig. S10: Basic design of the sample chamber**

This figure shows the basic design of the sample chamber. The carbon electrodes are attached to a simple microscopic glass slide with resin adhesive so that no medium can leak out of the chamber. The spacing of the electrodes determines the electric field strength within the chamber and should not exceed 5 mm, especially since otherwise the field strength (at 9V, the resulting field strength is 1800 V/m) may not be sufficient to reliably induce contraction.

The connection between electrodes and electrical wire contacting the MyoPulser (right) can be soldered, but must be embedded in synthetic resin to be watertight. Otherwise, contact with the medium during an experiment causes electrolysis of the copper leads and release of highly toxic metal ions into the medium. An additional cover glass can be used to prevent evaporation of the aqueous medium component during an experiment, that may increase ion concentrations or even drying of the sample, impacting experimental results.

Inverse microscopy is recommended but not mandatory using this type of chamber.

**Fig. S11: Construction of the ESP32-based MyoPulser – connection of LCD-display and sample chamber**

The upper part of this figure depicts the connection of the ESP32-pins to the pins of an 4x20-LCD-disply for proper control, including a rotary potentiometer (on the right) to adjust display brightness.

The lower part depicts connection of the sample chamber as well as the pins of the ESP32 that have to be connected to the “motor controller”-board.

As in **Fig. S3**, the 10 kΩ resistor (bottom right) replaces the sample chamber during measurement of the devices’ output with an oscilloscope, simulating a high impedance short circuit, but is NOT connected during an experiment at the same time as the sample chamber.

**Fig. S12: Further hardware of the ESP32-based MyoPulser**

Here, connection of further hardware to the ESP32-microcontroller is depicted in detail. Top left: the trigger of single pulses in “Monopulse-mode” of the MyoPulser, top right: the LED that indicates pulsing. Bottom left: the LED indicating data transfer between ESP32 and connected computer; bottom right: the LED indicating manual/pause mode of the device.

All compounds are connected via a 220 Ω resistor to limit current.

**Fig. S13: The ESP32-based MyoPulser (prototype using a breadboard)**

This image depicts the prototype of the ESP32-based MyoPulser, connected via breadboard and jump wires, without soldering. This setup can be used for test purposes or stationary operation, especially as it has limited transportability.

The main components of the setup are labeled in the figure.

**Fig. S14: The ESP32-based MyoPulser (proposal for a transportable setup)**

This image depicts a transportable form of the ESP32-based MyoPulser, where the single components are soldered on a board. This suggested setup is mechanical more stable, less susceptible to loose contacts and much clearer than the version depicted in **Fig. S13**.

The individual components are labeled, the connecting cables are located on the underside of the soldered board, protected by a base plate (plastic, about 2 mm thick) kept at a distance of about 5 mm by spacer screws.

*IMPORTANT*: The push button for manual pulsing (“Monopulse-trigger”) can also be replaced by an external electronic device which serves as trigger for the MyoPulser.

**Fig. S15: “Arduino-IDE”-settings for the ESP32-based MyoPulser**

This figure displays the setting applied to the “Arduino-IDE” when the control software is loaded on the ESP32.

**Fig. S16: The user interface of the ESP32-based MyoPulser**

For proper function, the ESP32-based MyoPulser must be connected to a computer running the appropriate control software. This is programmed in Java and runs on the free software platform “*Processing*” (version 4.0 beta 6). “*Processing*” generates the necessary user interface. The settings can be changed in predefined ranges and are only then transferred to the ESP32 via mouse click. In this way, there is no change during the resetting as when using analog components such as rotary potentiometers. Pulse times are always displayed in red, pauses in blue. Pulse times are always displayed in red, gaps in blue. The currently applied pulse type (bottom left) is highlighted in color, to the right of it the pulse shape is displayed as a graphic. Furthermore, detailed information such as total experimental time, applied pulses at the current settings as well as total pulses and repolarization time (for polyphasic pulsing only) are displayed in real time. Also available is a simple stopwatch with split time (bottom right).

All these data are simultaneously written to a file on the hard disk of the connected computer.

The single elements and features of the user interface are labelled in this figure.

**Fig. S17: Display and pulse-modes of the ESP32-based MyoPulser**

Due to the lack of analog components, the ESP32-based MyoPulser does not need a “Hardware-Test-Mode”, and consequently has only three different operating states: “Normal-Mode”, “Random-Mode” and “Manual-Mode”. In the according modes, the most important settings are shown on the display, including current mode, pulse and gap times, applied pulse type and phase. The “Manual- Mode” also serves as a pause mode, which interrupts automatic pulsing.

**Fig. S18: Connecting the ESP32 to a COM-port via the software “*Processing*”**

Identification and check of the detected COM-ports when starting the software “*Processing*”. In this case, only the COM-port “COM8” was identified (red rectangle) and responded property to an identification phrase that has been sent to the MyoPacer. After this, the MyoPacer is connected properly and can be used via the interface.
