# Supplementary figures and images for: The MyoPulser field stimulator, a do it yourself programmable electronic pacemaker for contracting cells and tissues
Source: Sci Rep. 2023 Feb 11;13:2461. doi: 10.1038/s41598-023-29145-3 (PMC9922332; doi:10.1038/s41598-023-29145-3)

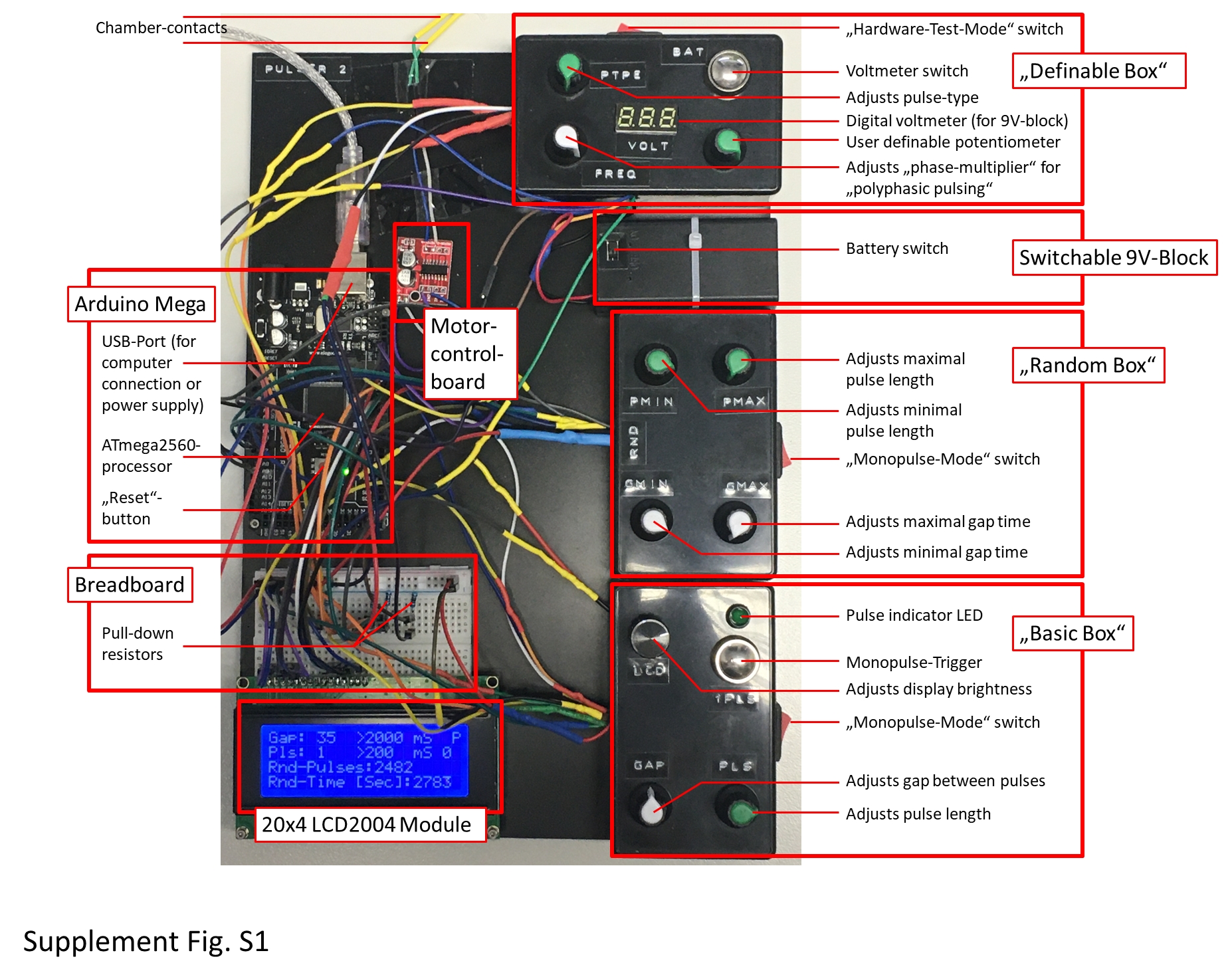

Supplement: Supplementary file 2 — Supplementary Figure S1. [file 41598_2023_29145_MOESM2_ESM.jpg]

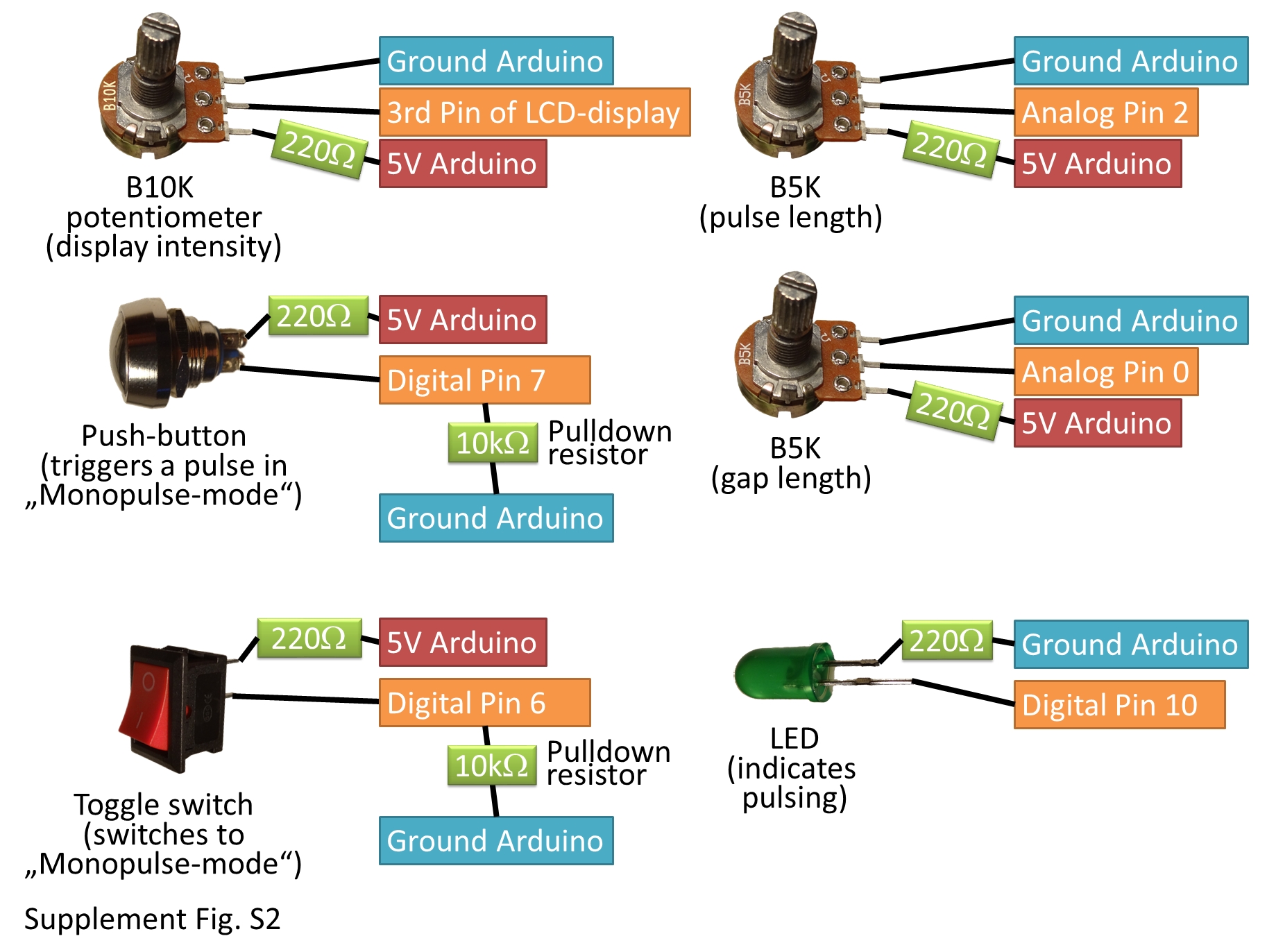

Supplement: Supplementary file 3 — Supplementary Figure S2. [file 41598_2023_29145_MOESM3_ESM.jpg]

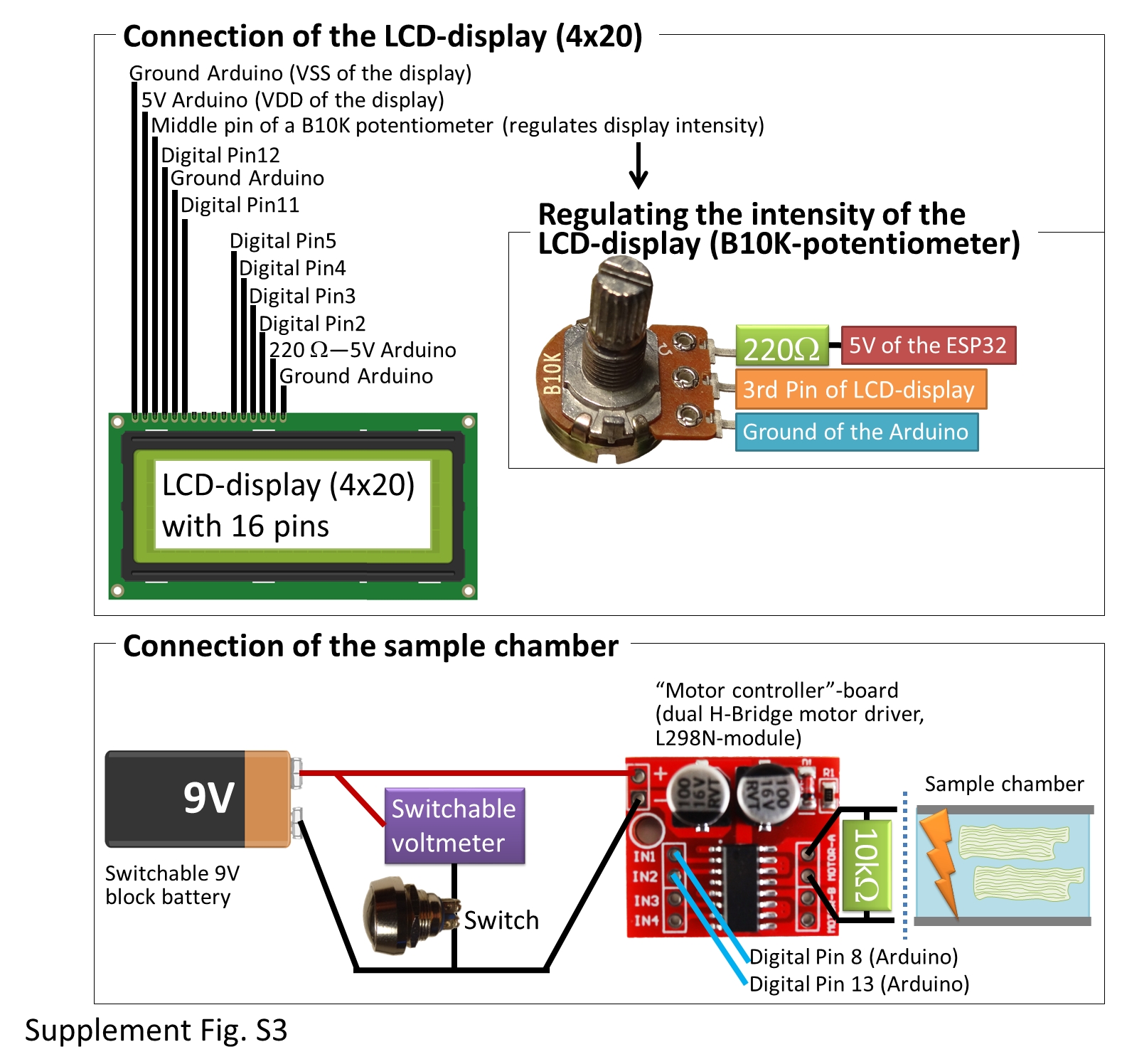

Supplement: Supplementary file 4 — Supplementary Figure S3. [file 41598_2023_29145_MOESM4_ESM.jpg]

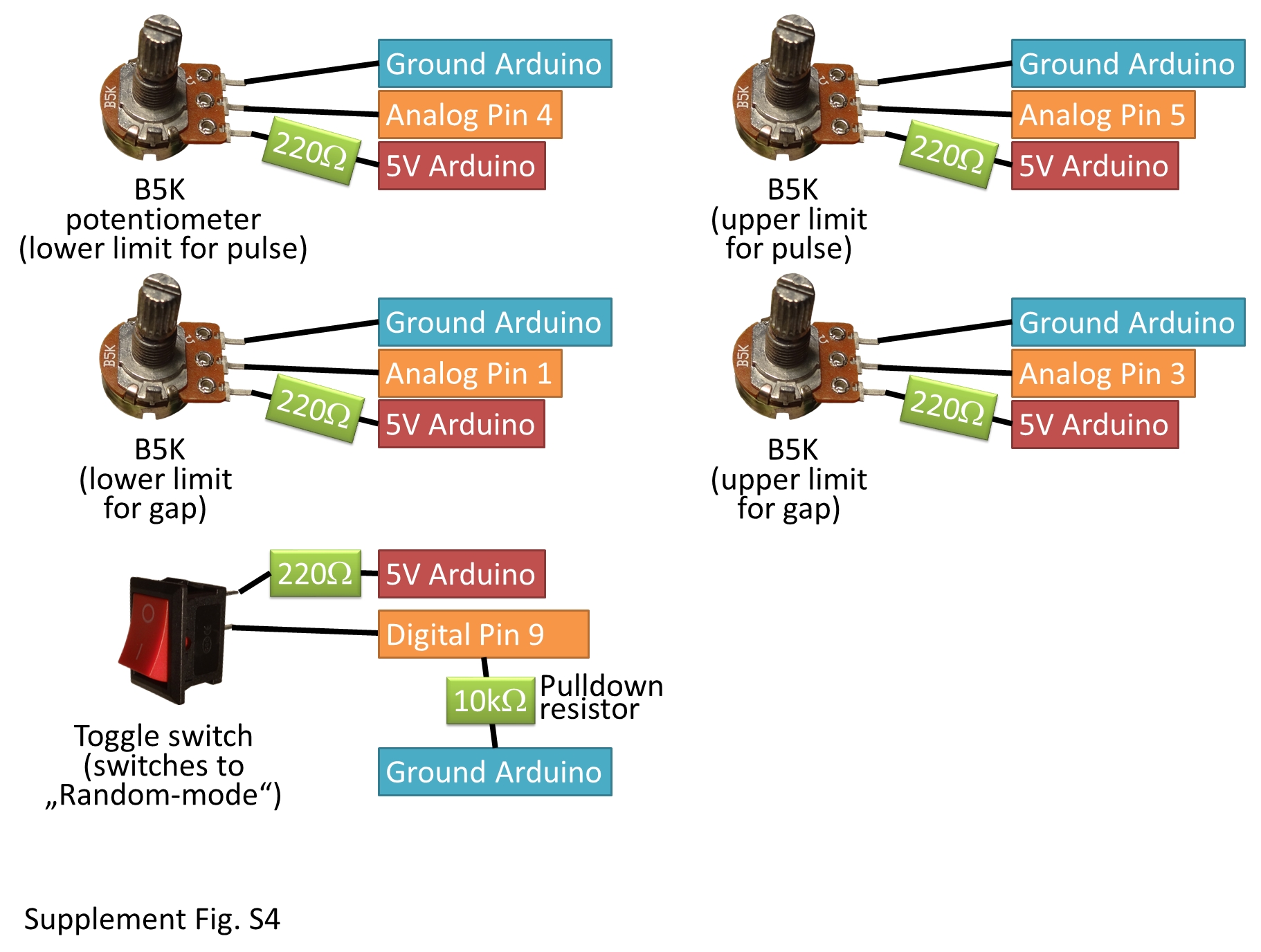

Supplement: Supplementary file 5 — Supplementary Figure S4. [file 41598_2023_29145_MOESM5_ESM.jpg]

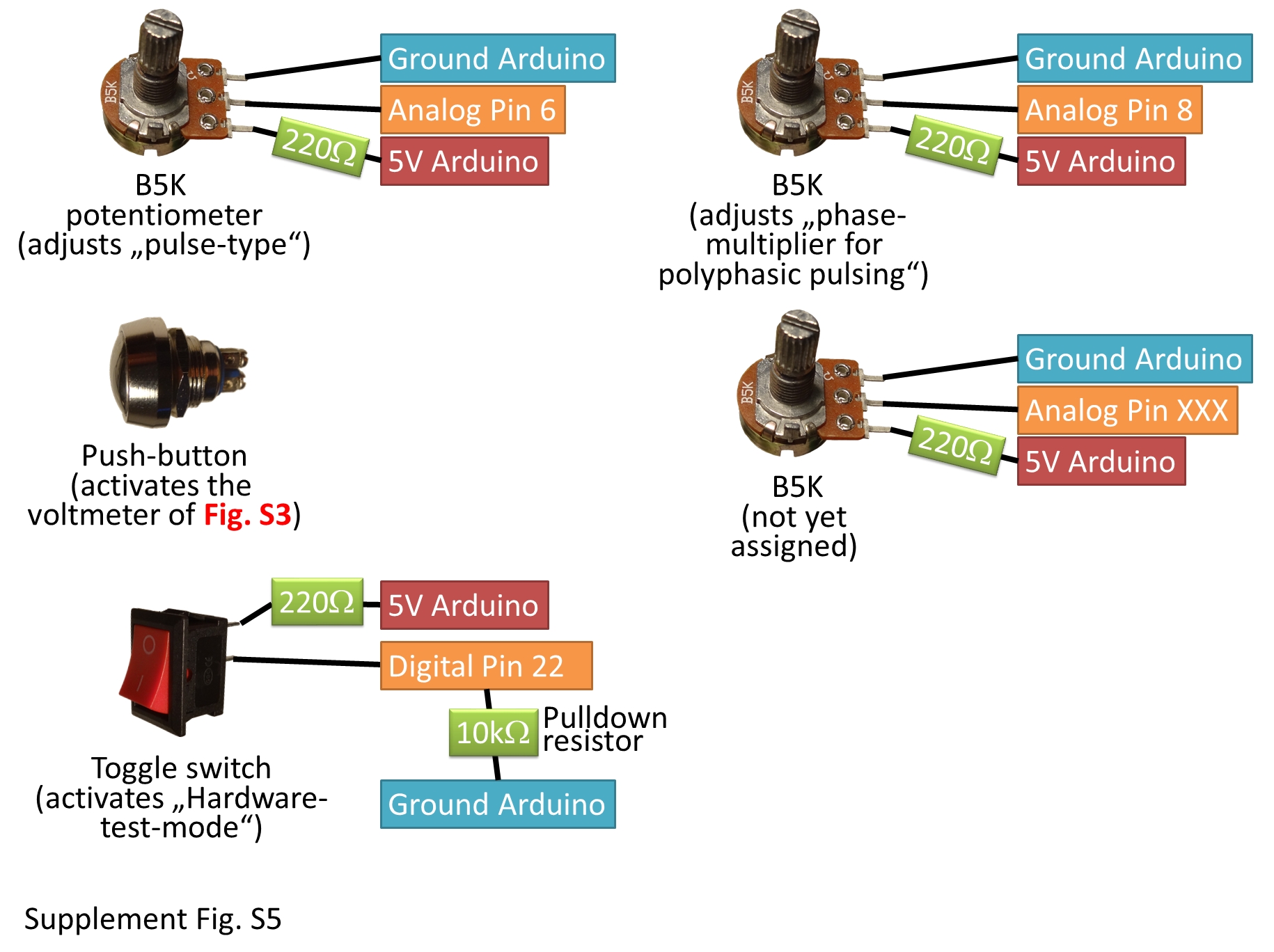

Supplement: Supplementary file 6 — Supplementary Figure S5. [file 41598_2023_29145_MOESM6_ESM.jpg]

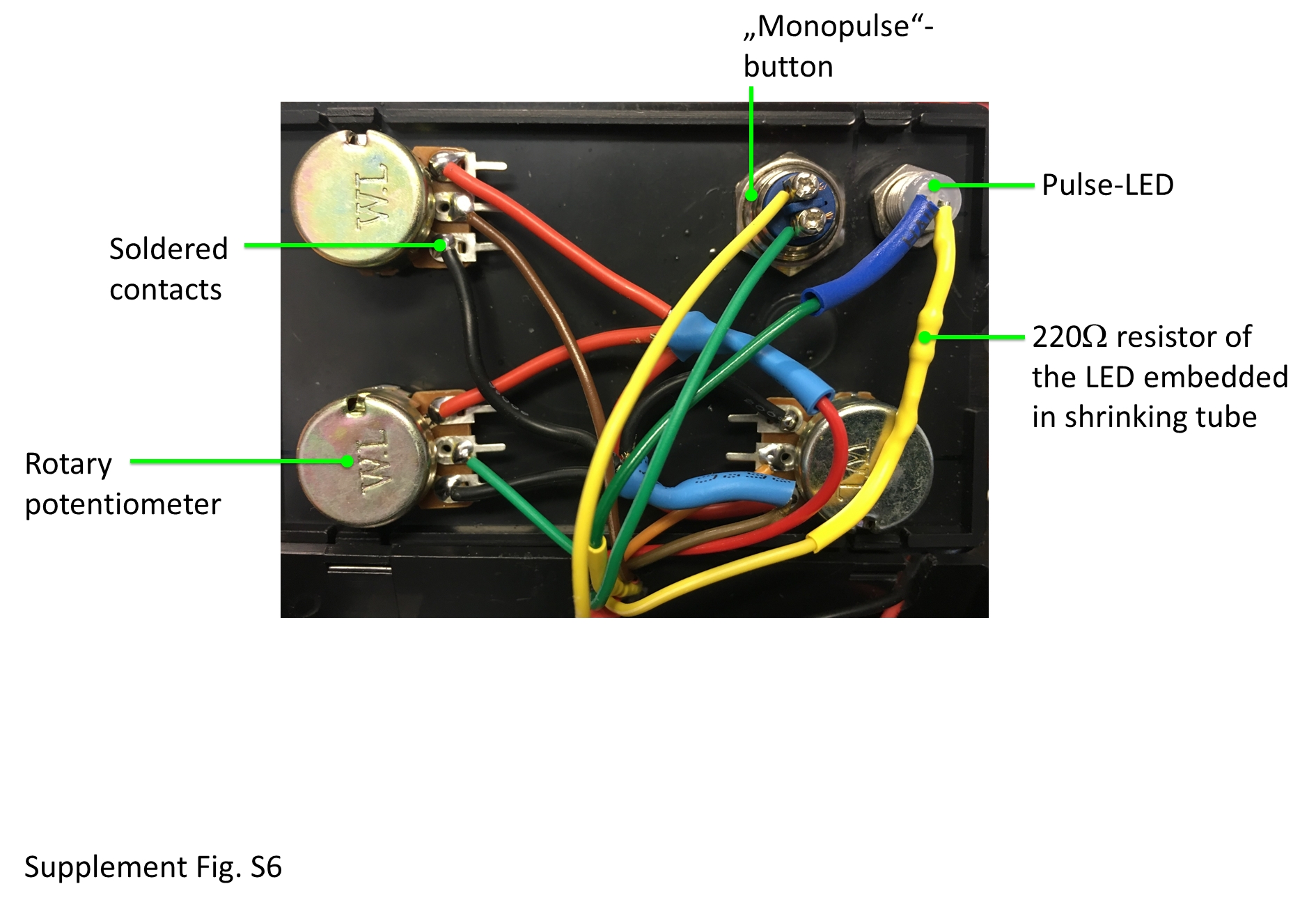

Supplement: Supplementary file 7 — Supplementary Figure S6. [file 41598_2023_29145_MOESM7_ESM.jpg]

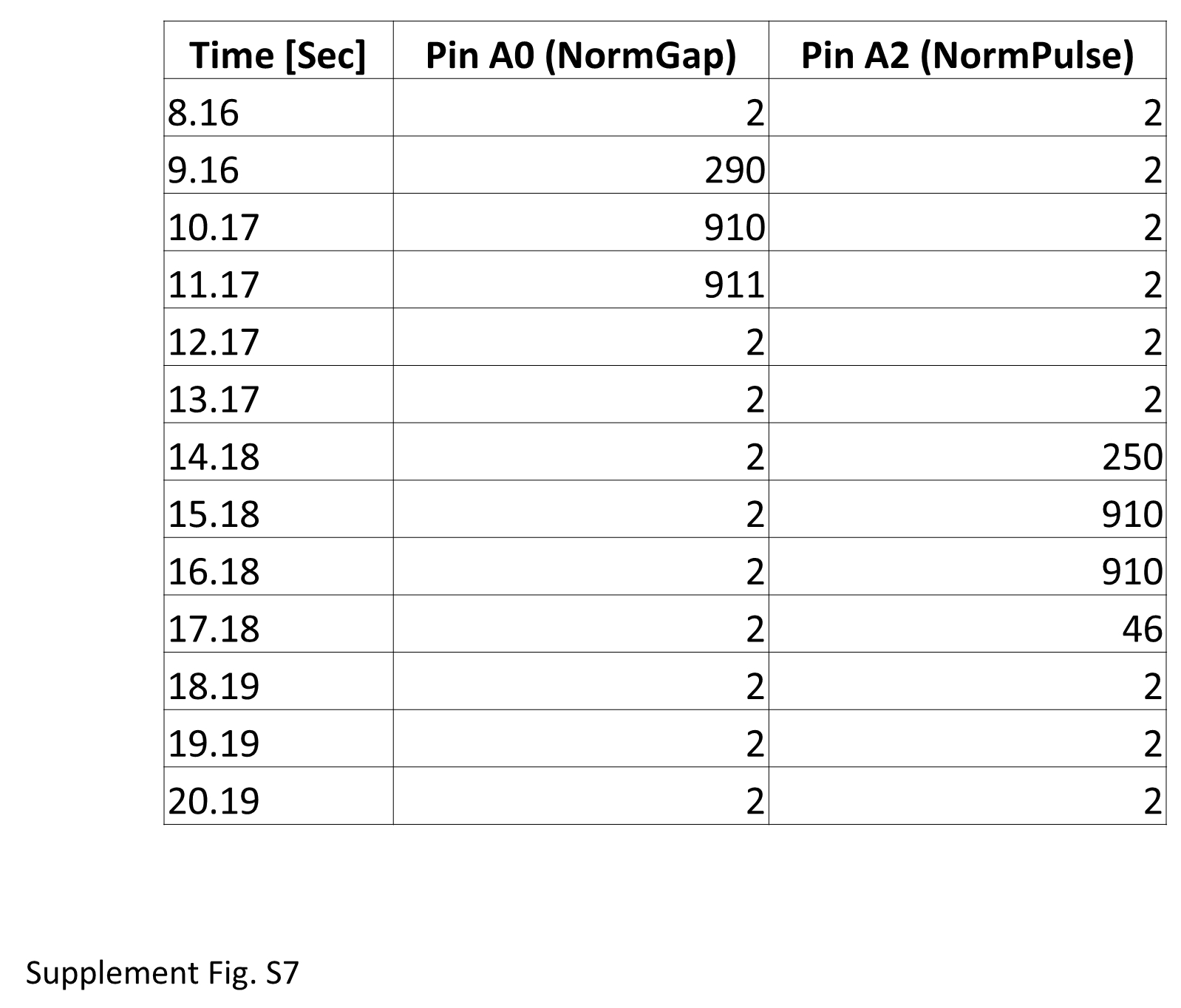

Supplement: Supplementary file 8 — Supplementary Figure S7. [file 41598_2023_29145_MOESM8_ESM.jpg]

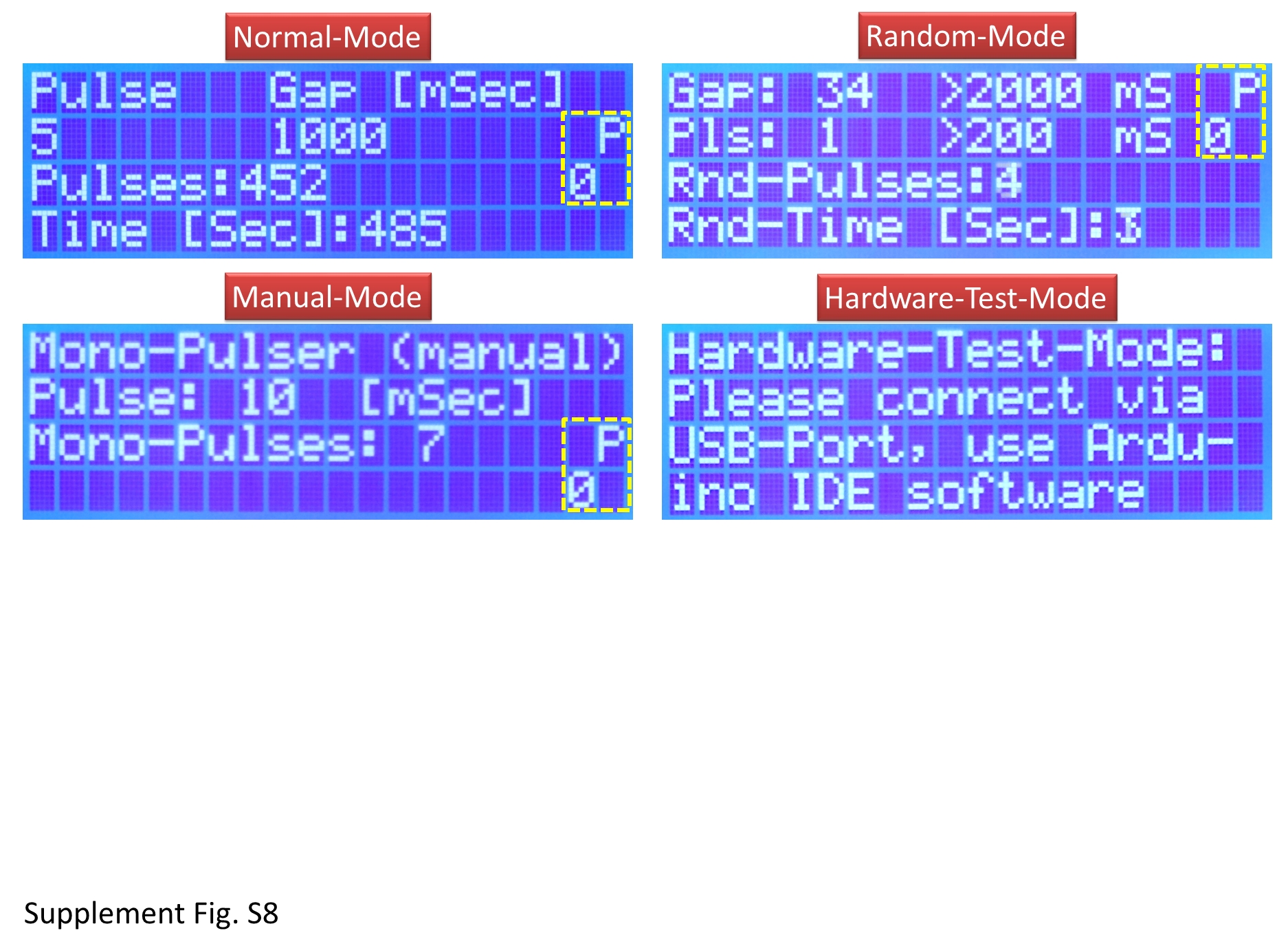

Supplement: Supplementary file 9 — Supplementary Figure S8. [file 41598_2023_29145_MOESM9_ESM.jpg]

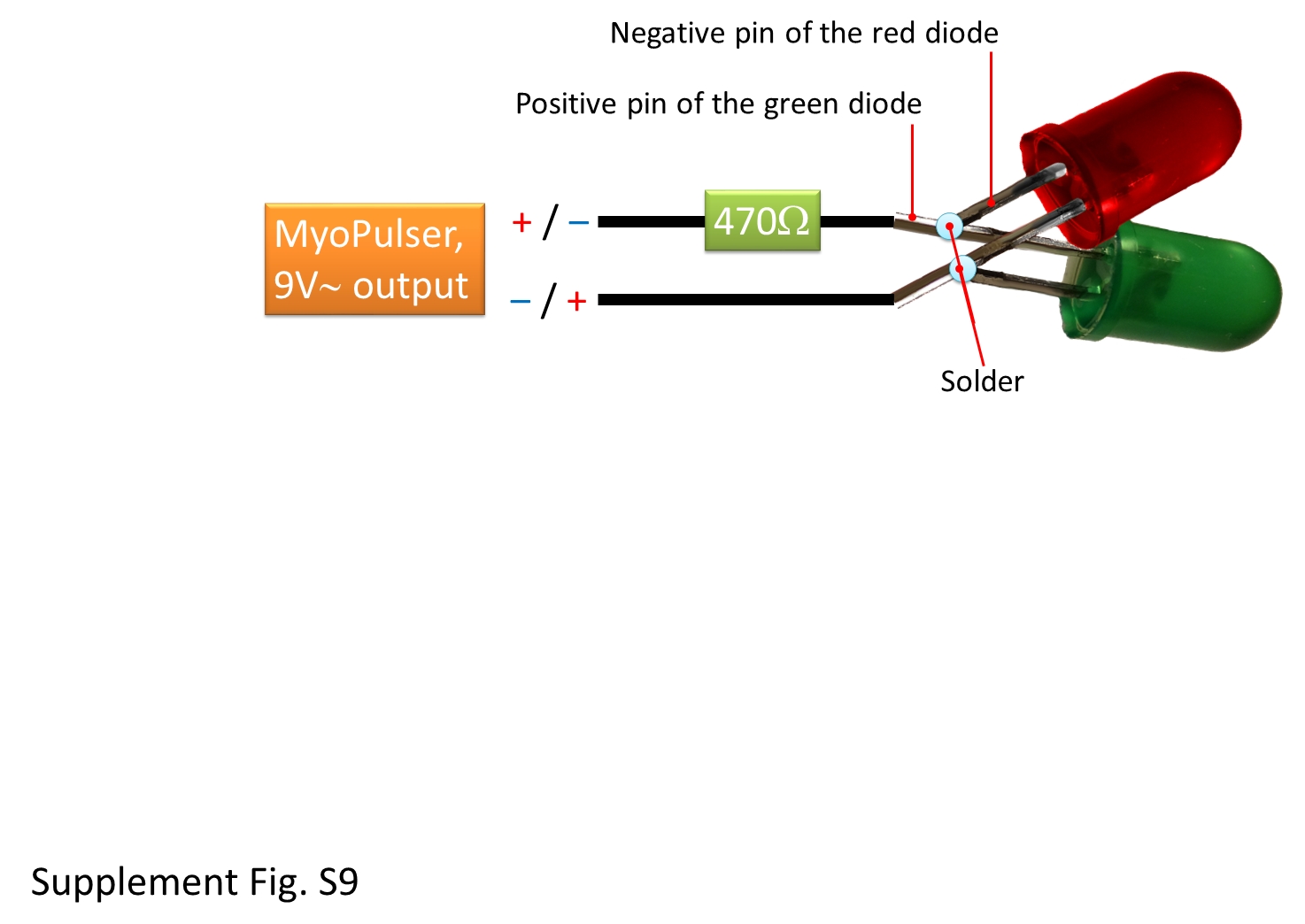

Supplement: Supplementary file 10 — Supplementary Figure S9. [file 41598_2023_29145_MOESM10_ESM.jpg]

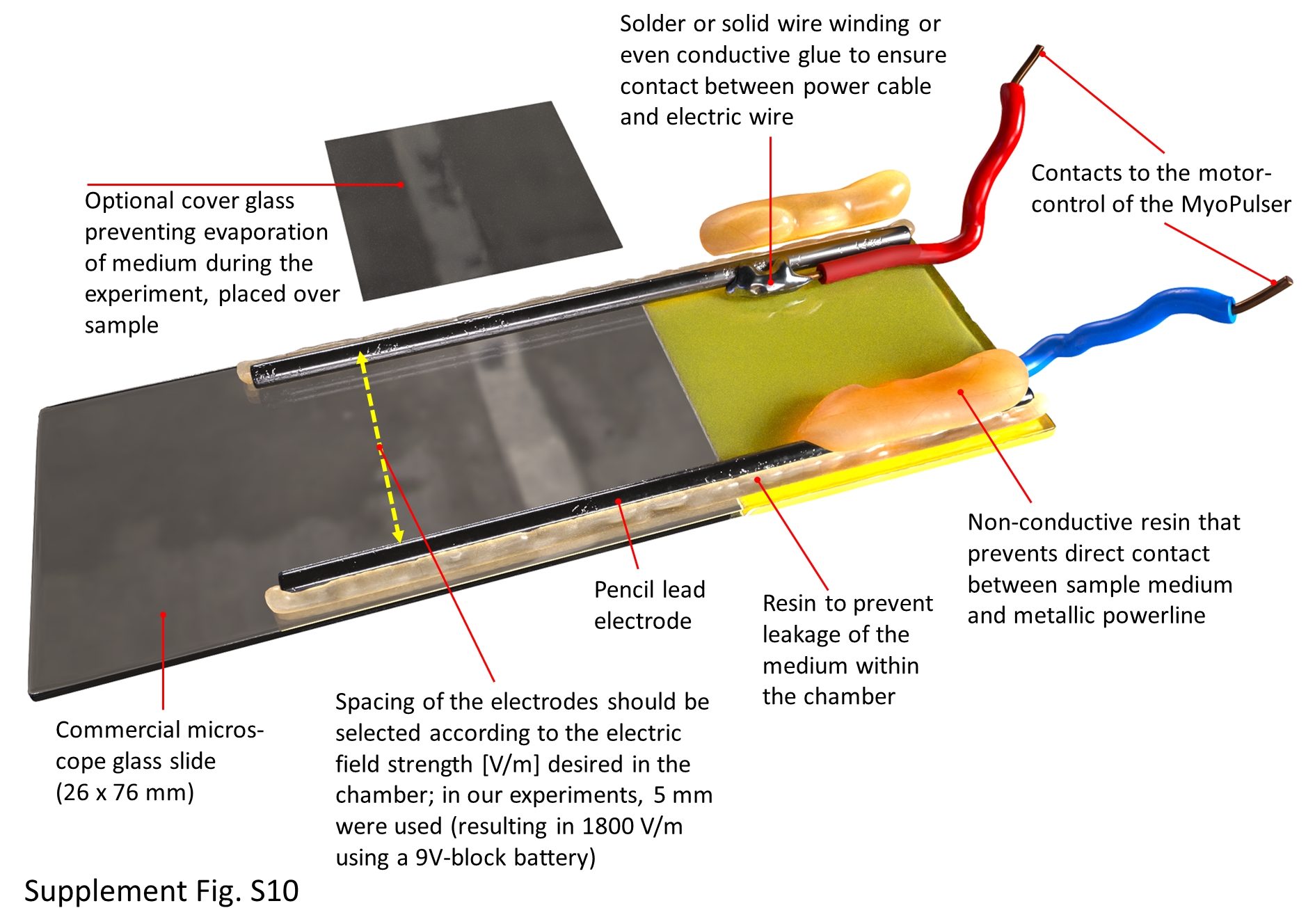

Supplement: Supplementary file 11 — Supplementary Figure S10. [file 41598_2023_29145_MOESM11_ESM.jpg]

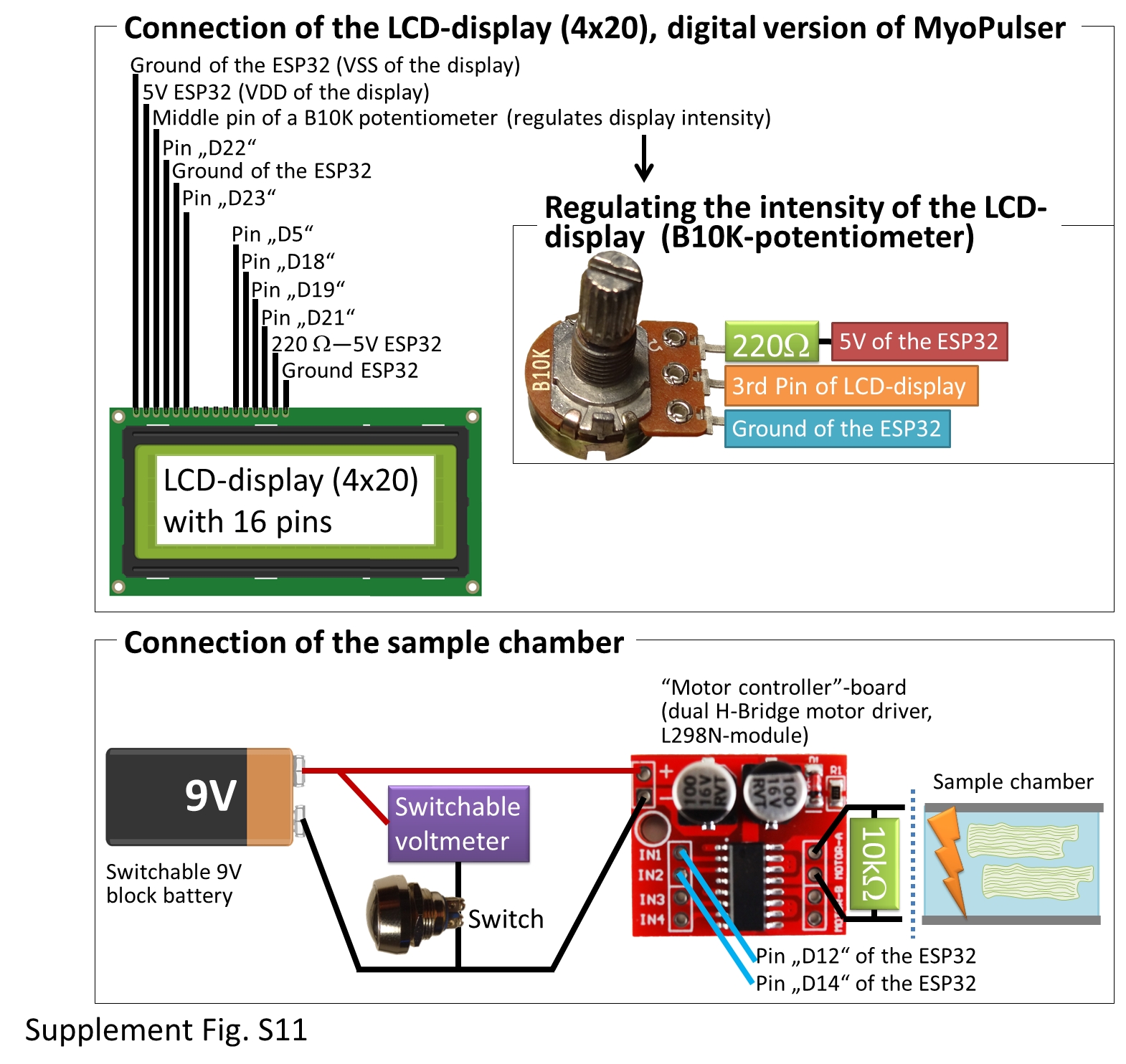

Supplement: Supplementary file 12 — Supplementary Figure S11. [file 41598_2023_29145_MOESM12_ESM.jpg]

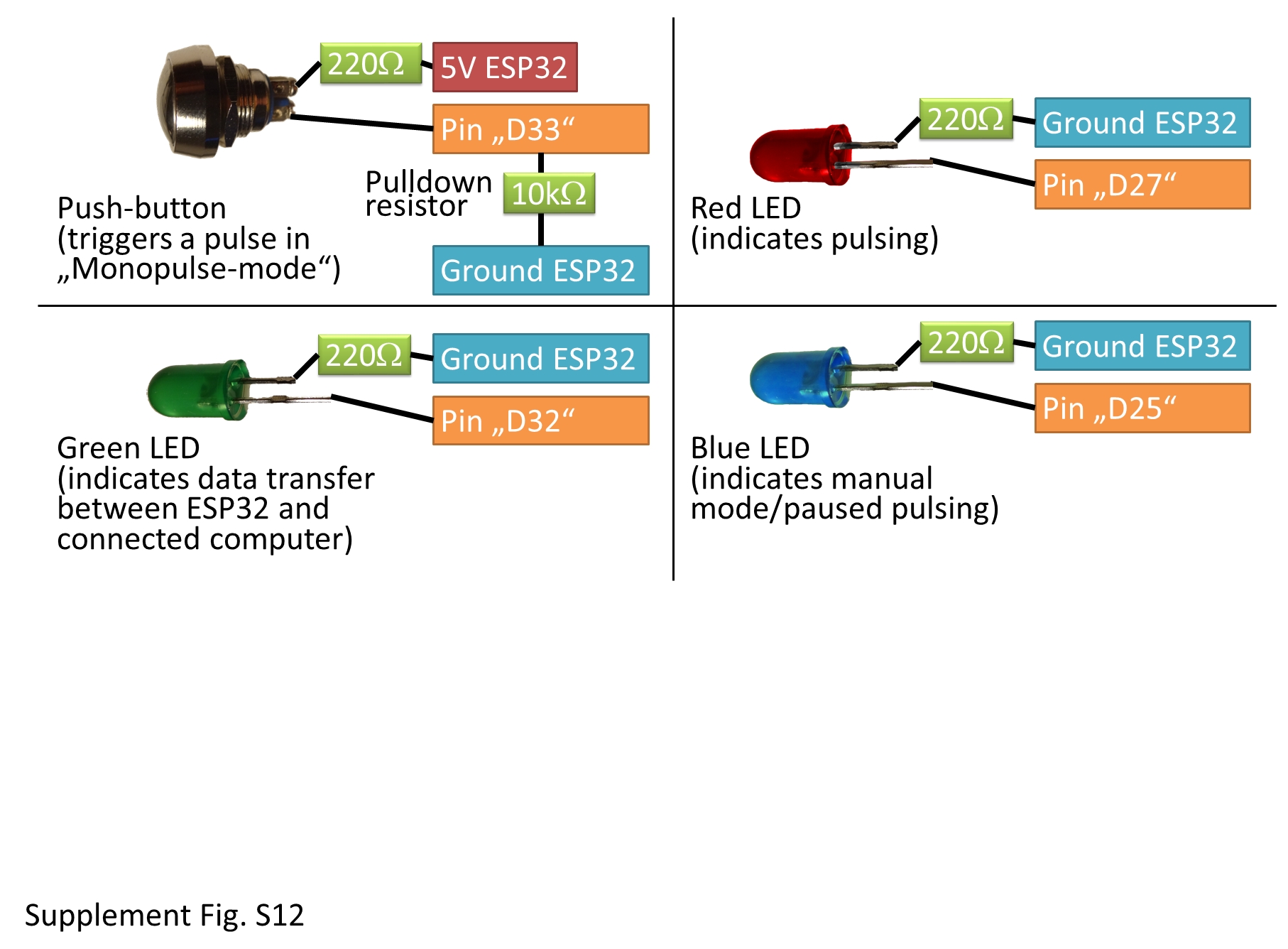

Supplement: Supplementary file 13 — Supplementary Figure S12. [file 41598_2023_29145_MOESM13_ESM.jpg]

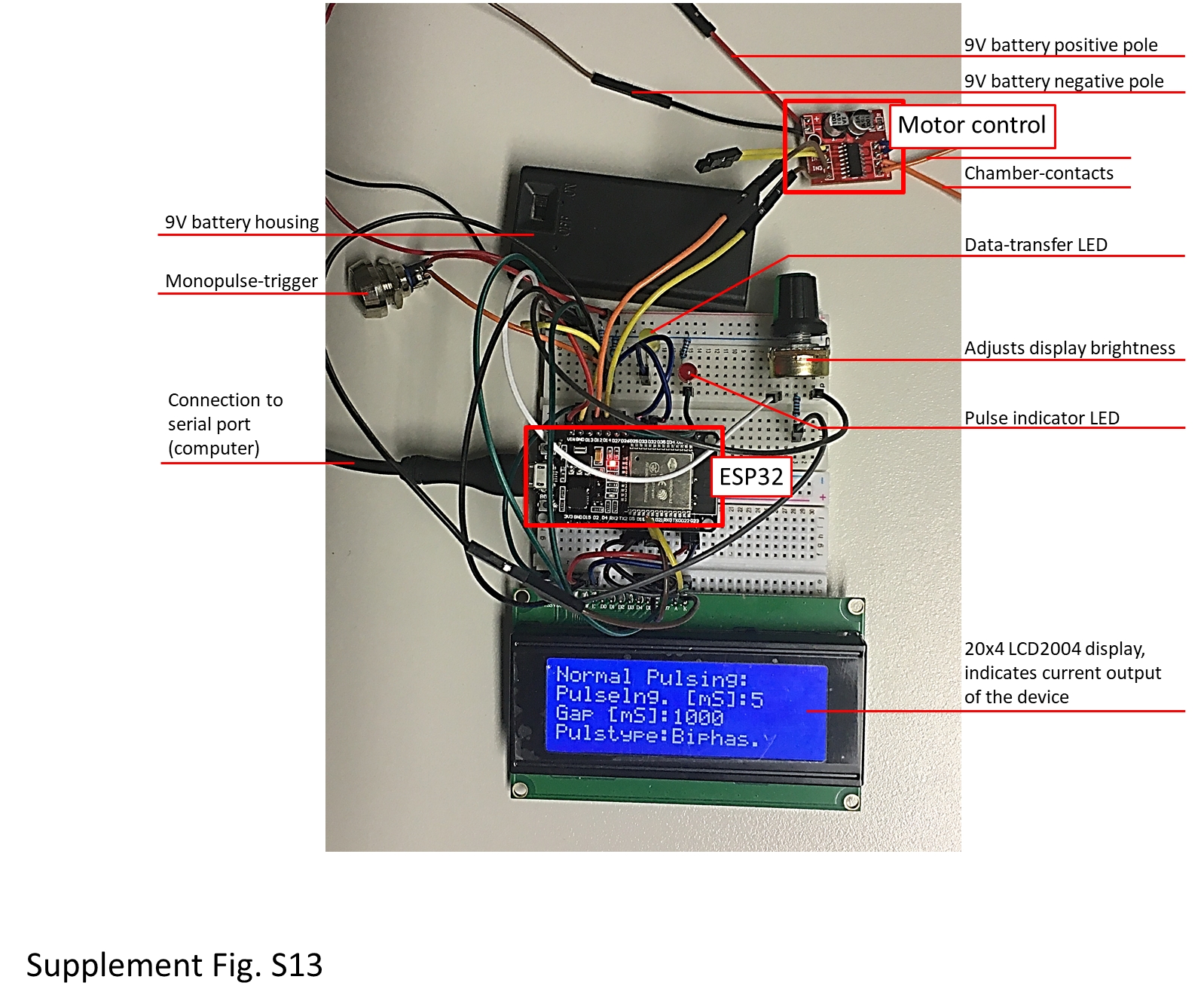

Supplement: Supplementary file 14 — Supplementary Figure S13. [file 41598_2023_29145_MOESM14_ESM.jpg]

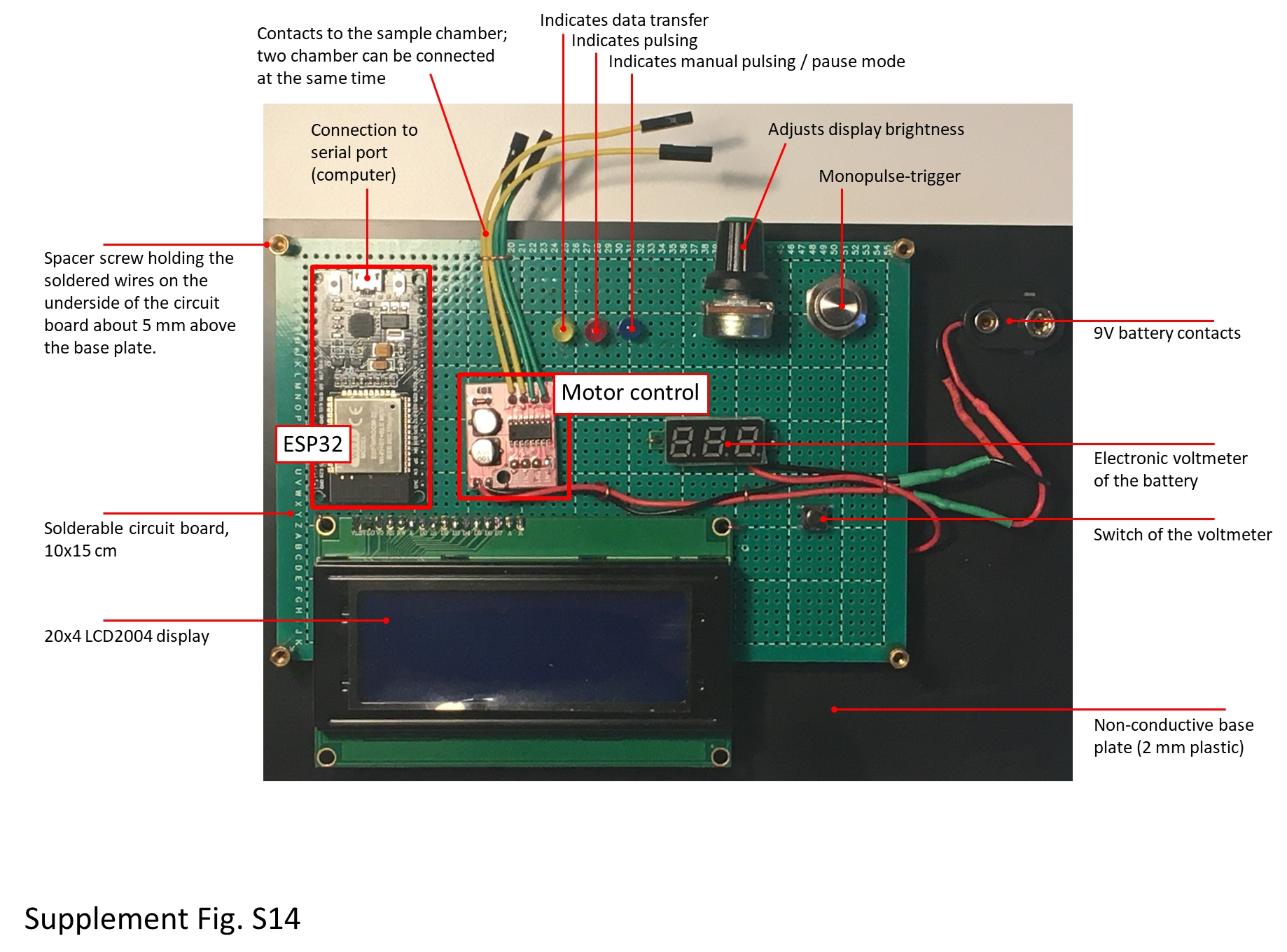

Supplement: Supplementary file 15 — Supplementary Figure S14. [file 41598_2023_29145_MOESM15_ESM.jpg]

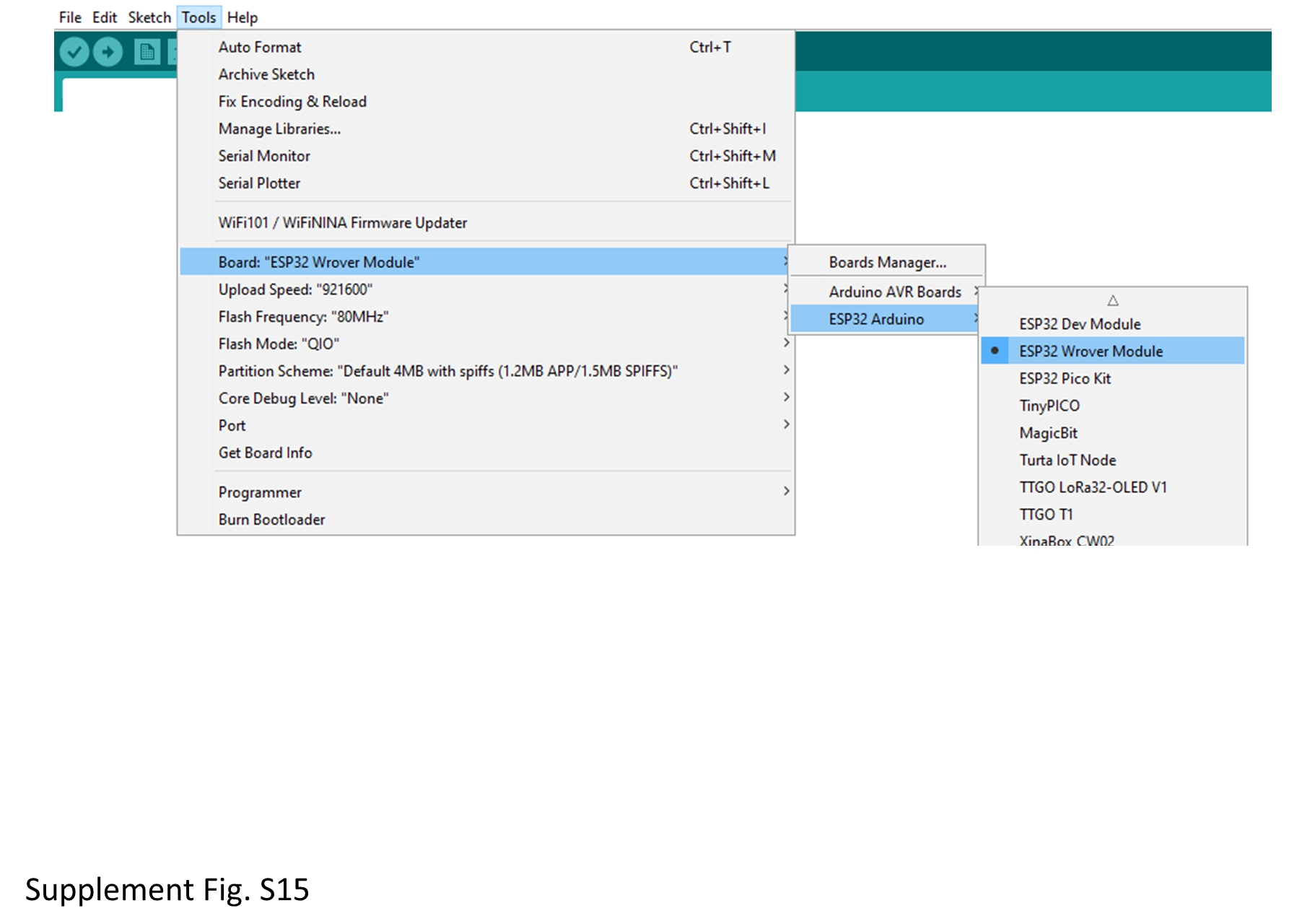

Supplement: Supplementary file 16 — Supplementary Figure S15. [file 41598_2023_29145_MOESM16_ESM.jpg]

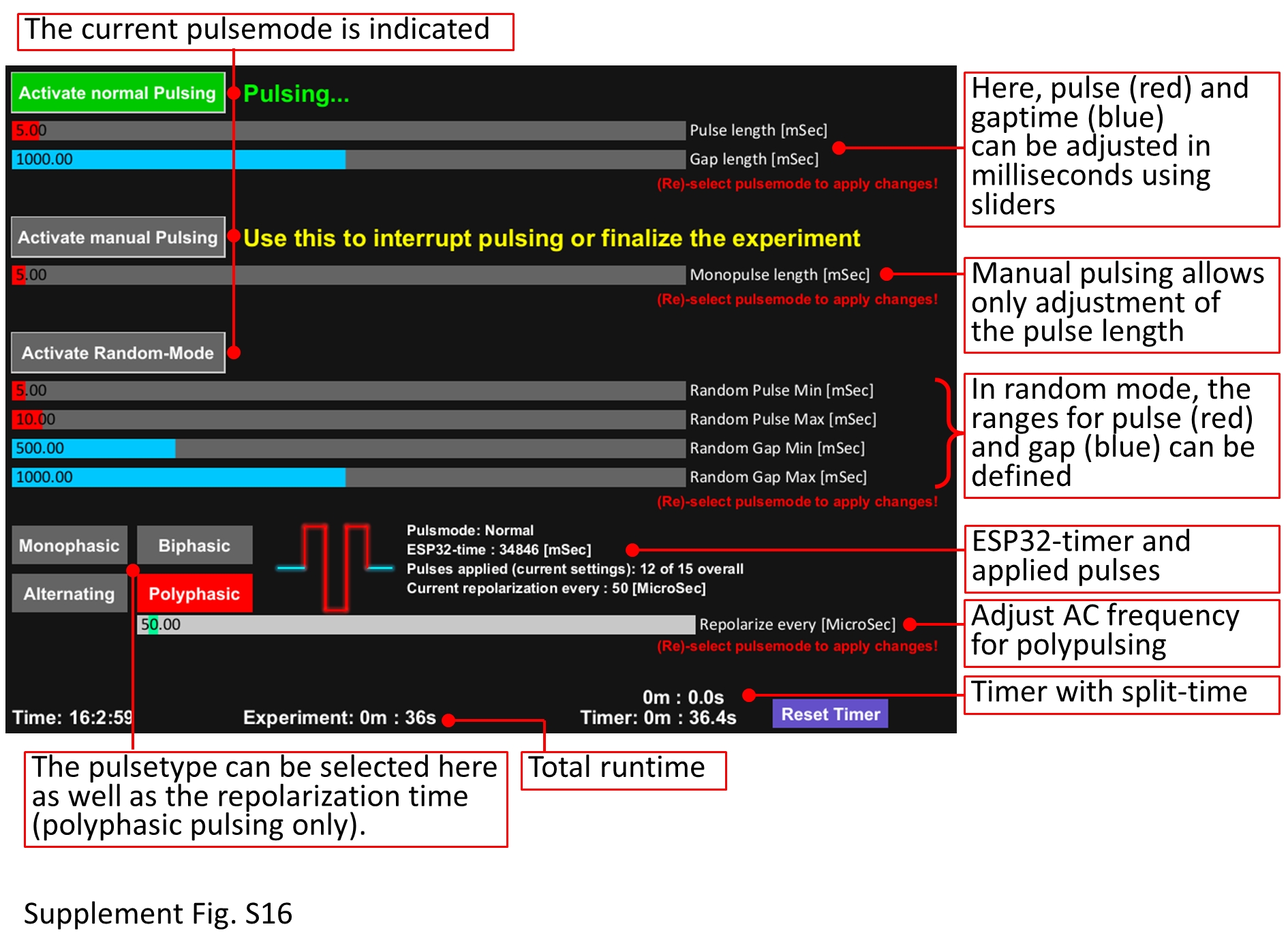

Supplement: Supplementary file 17 — Supplementary Figure S16. [file 41598_2023_29145_MOESM17_ESM.jpg]

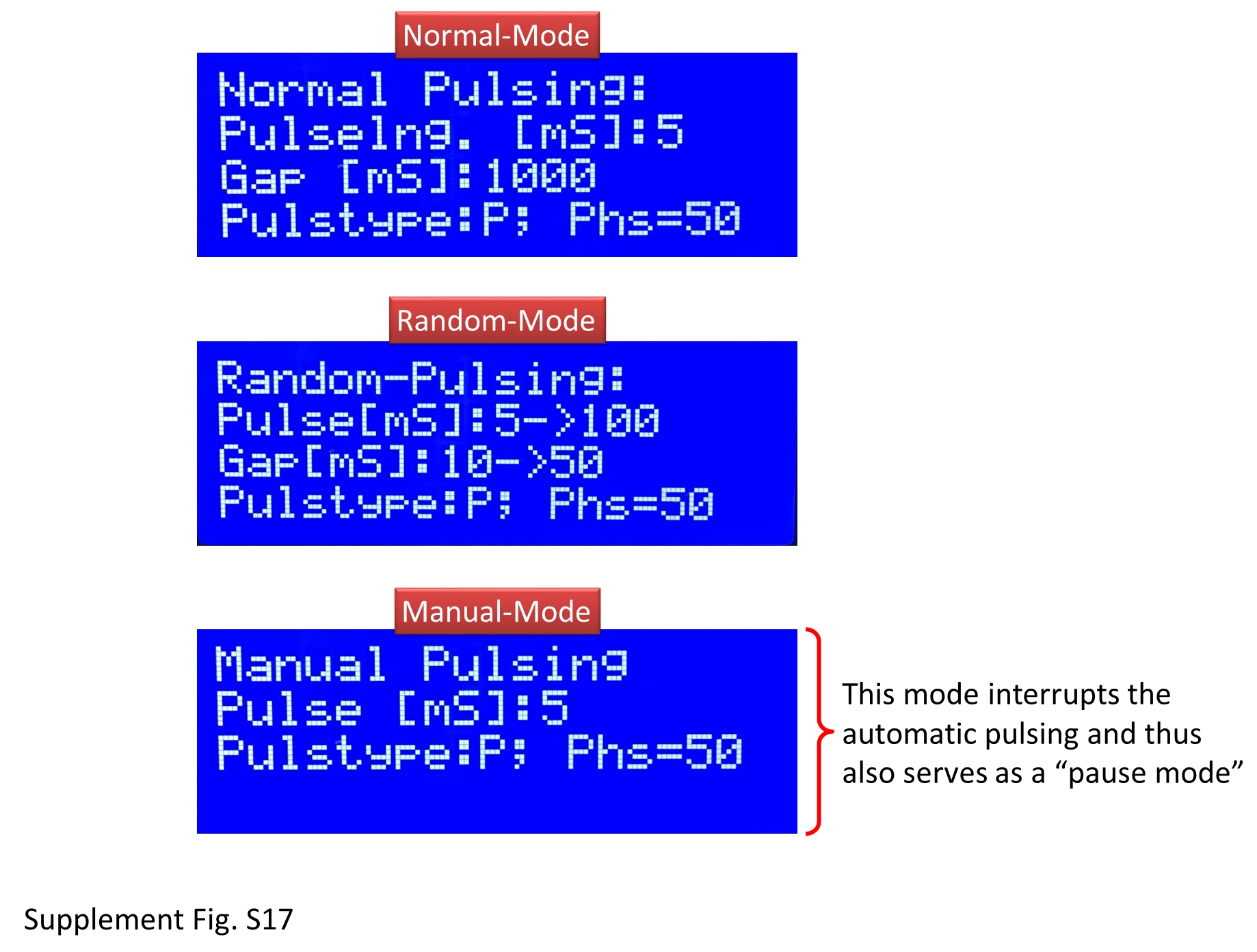

Supplement: Supplementary file 18 — Supplementary Figure S17. [file 41598_2023_29145_MOESM18_ESM.jpg]

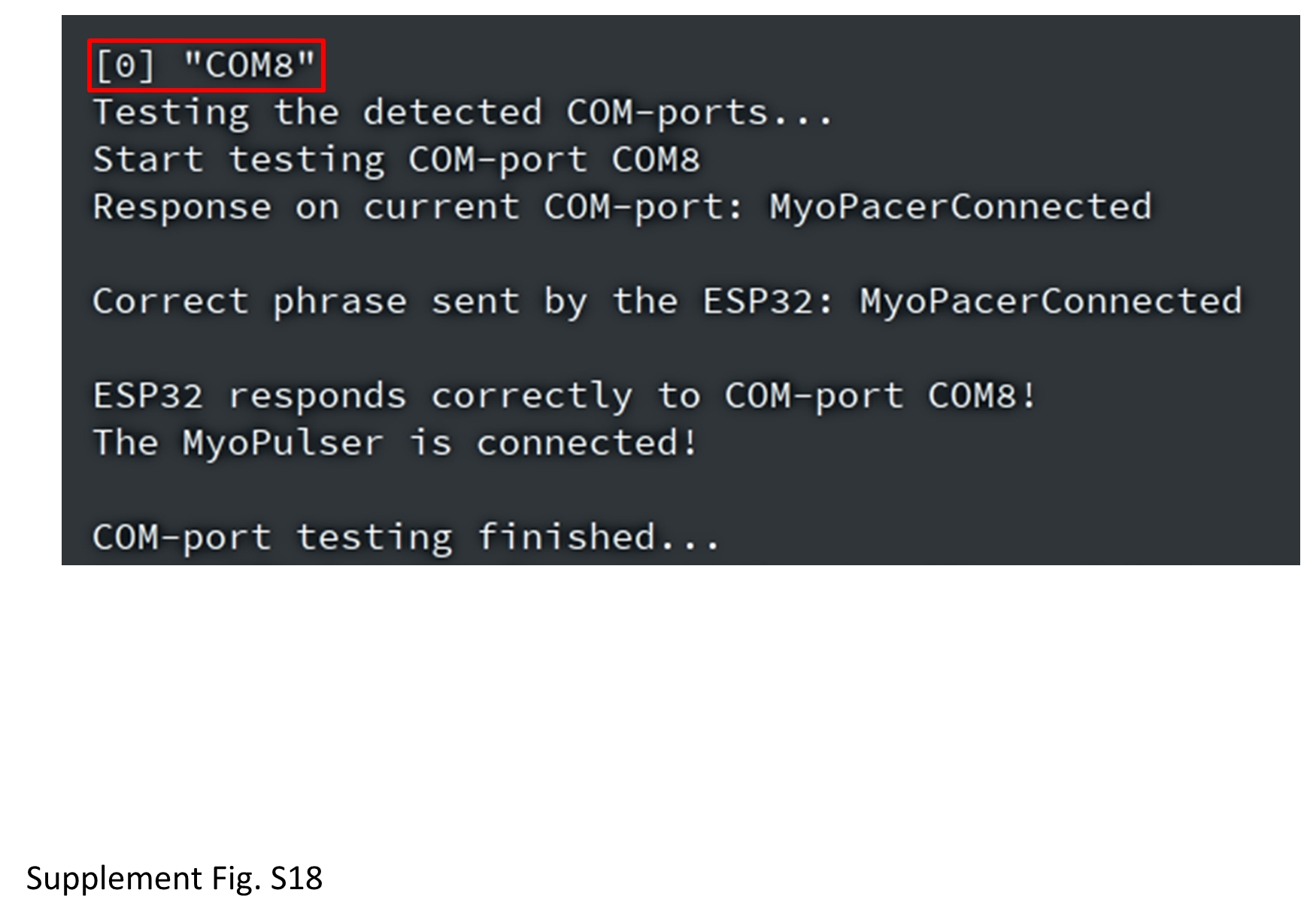

Supplement: Supplementary file 19 — Supplementary Figure S18. [file 41598_2023_29145_MOESM19_ESM.jpg]

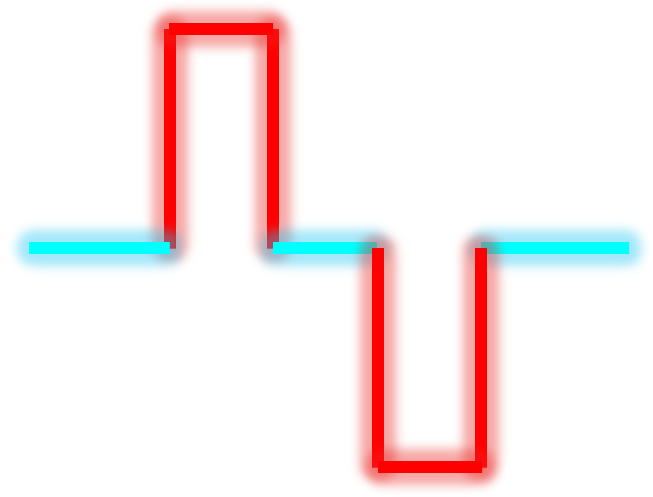

Supplement: Supplementary file 20 — Supplementary Information 2. [file 41598_2023_29145_MOESM20_ESM.zip › alternating.png]

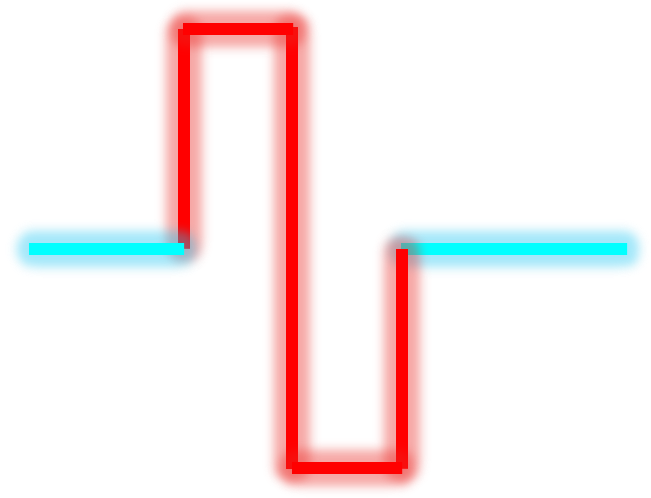

Supplement: Supplementary file 20 — Supplementary Information 2. [file 41598_2023_29145_MOESM20_ESM.zip › biphasic.png]

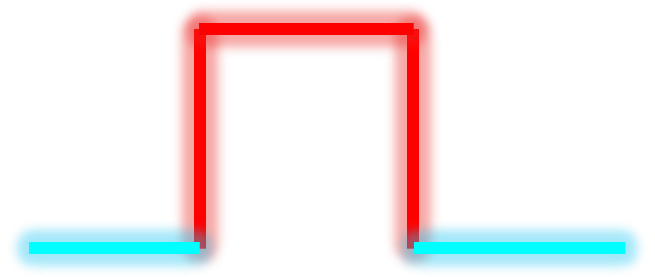

Supplement: Supplementary file 20 — Supplementary Information 2. [file 41598_2023_29145_MOESM20_ESM.zip › monophasic.png]

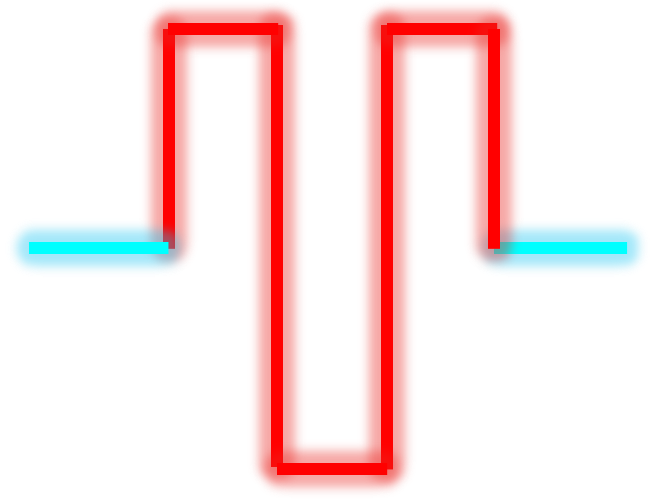

Supplement: Supplementary file 20 — Supplementary Information 2. [file 41598_2023_29145_MOESM20_ESM.zip › polyphasic.png]
